# Frog vocal sacs-inspired soft acoustic system with continuously tunable resonance for sound emission and stethoscopic sensing

**DOI:** 10.1126/sciadv.adz5930

**Published:** 2025-12-17

**Authors:** Chuting Liu, Peiyan Dong, Jiantao Wang, Zhikang Deng, Jinan Luo, Chang Liu, Jingzhi Wu, Kaiyuan Tan, Jiajun Pan, Rongkuan Han, Yuanfang Li, Lvjie Chen, Xinyi Qu, Jianfeng Ma, Qinghong Zhou, Bojun Yan, Yu Ran, Dalun Rong, Jianping Jiang, Bo Li, Tian-Ling Ren, Jianhua Zhou, Yancong Qiao

**Affiliations:** ^1^ School of Biomedical Engineering, Shenzhen Campus of Sun Yat-sen University, No. 66, Gongchang Road, Guangming District, Shenzhen, Guangdong 518107, China.; ^2^ Key Laboratory of Sensing Technology and Biomedical Instruments of Guangdong Province, School of Biomedical Engineering, Sun Yat-sen University, Guangzhou 510275, China.; ^3^ Dongguan Aopu New Audio Technology Co. Ltd., No. 5, Keji 2nd Road, Songshan Lake Park, Dongguan, Guangdong 523000, China.; ^4^ School of Aeronautics and Astronautics, Shenzhen Campus of Sun Yat-sen University, No. 66, Gongchang Road, Guangming District, Shenzhen, Guangdong 518107, China.; ^5^ School of Civil Engineering, Hunan University of Technology, Zhuzhou, Hunan 412007, China.; ^6^ Cardiovascular Surgery, Seventh Affiliated Hospital of Sun Yat-sen University, Shenzhen 518107, China.; ^7^ School of Integrated Circuits and Beijing National Research Center for Information Science and Technology (BNRist), Tsinghua University, Beijing 100084, China.

## Abstract

To overcome limitations of flexible thermoacoustic devices in low-frequency emission and sensing, we present a resonance-adjustable graphene sound device (RAGSD) inspired by frog vocal sacs. Integrating laser-induced graphene (LIG) with a deformable cavity, RAGSD provides continuous tuning from 922.12 to 1762.90 hertz. A dynamic, continuously tunable electro-mechano-acoustical model explains the mechanism and predicts frequency with $R^{2}=0.990$ . In emission, a 25.34-decibel sound pressure level gain was observed at resonance, enabling controllable, frequency-selective voice amplification for personalized output. In sensing, the LIG piezoresistive readout delivers sensitive transduction, while resonance matching amplifies weak, high-frequency cardiac sounds. Wearable tests on healthy volunteers and patients recorded clear S1/S2 and pathological murmurs. Integrated with AuscNet-H, a deep learning algorithm designed for heart sound classification, the system achieved 99.375% accuracy across four clinical classes. Under inflation, no false negatives occurred, and misclassifications among similar diseases were fewer than with a commercial electronic stethoscope. These results demonstrate a practical path toward intelligent, wearable auscultation.

## Teaser

Frog-inspired, soft, continuously tunable acoustics are designed for personalized voice and wearable auscultation.

## INTRODUCTION

The thermoacoustic effect arises from the coupling between heat conduction and entropy variation in oscillating fluid boundary layers, where thermal perturbations induce pressure oscillations that generate or amplify sound waves (*1*–*3*). In thermoacoustic sound sources (TASSs), ac applied to a conductive material produces Joule heating and temperature oscillations, which drive acoustic emission in the surrounding medium (*4*, *5*). Graphene has emerged as a highly promising material for TASSs due to its outstanding thermal conductivity (*6*), large specific surface area (*7*), high electrical conductivity, and excellent mechanical flexibility (*8*–*10*). These properties enable efficient and rapid thermal exchange, allowing graphene-based thermoacoustic devices to achieve broadband acoustic response theoretically spanning from subaudio to ultrasonic frequencies (*11*–*13*). In addition, the simplicity of the thermoacoustic mechanism enables sound generation without relying on macroscopic mechanical vibrations of rigid components, reducing wear-prone interfaces (*4*, *14*, *15*). These combined advantages make graphene TASSs attractive for integration into miniaturized, flexible, and wearable acoustic systems (*16*, *17*). However, despite the considerable promise of graphene for TASSs, existing graphene TASSs still exhibit limitations in sound pressure level (SPL) performance, particularly in the low-frequency range around 1 kilohertz (kHz) (*18*–*24*), which is a critical band that encompasses voice signals, heart sounds, and lung sounds. Table 1 compares the normalized SPL at 1 kHz for representative graphene TASSs, measured 1 cm from the microphone under 1-W input power. The observed SPL limitations at this frequency constrain their applicability in scenarios requiring accurate perception and transmission of low-frequency acoustic signals, such as electronic stethoscopes, voice assistive systems, and other clinical acoustic devices. Moreover, most flexible acoustic systems still separate emission and sensing (*25*–*29*), limiting integration and adaptability, and offer fixed bands without continuous alignment to the on-body monitored target or algorithm-coupled closed-loop operation (table S1) (*30*–*38*).

**Table 1.** Normalized SPL comparison of graphene TASSs and RAGSD. The boldfaced values correspond to the data obtained from this work. PI, polyimide; LSG, laser-scribed graphene; PET, polyethylene terephthalate; rGO, reduced graphene oxide; GrF, graphene foam; PU, polyurethane.

Name	Materials	Substrate	Normalized SPL at 1 kHz decibel (dB)	Reference
LIG artificial throat	LIG	PI	41.08	(*18*)
Graphene loudspeakers	LSG	PET	33.74	(*19*)
Graphene earphone	LSG	PET	33.98	(*20*)
rGO sound-emitting device	rGO	PET	48.98	(*21*)
GrF device	GrF	PU foam	36.02	(*22*)
Stretchable graphene device based on the thermal-acoustic effect	Graphene conductive slurry	A cotton/Spandex composite textile	20	(*23*)
Graphene artificial throat	LSG	PU thin film	40	(*24*)
**RAGSD**	**LIG**	**PI**	**59.73**	**This work**

To overcome these problems, researchers have introduced resonant cavities into TASSs to enhance SPL at low frequencies. Resonant cavities can amplify sound pressure (SP) in specific frequency ranges, and when the sound wave frequency matches the cavity’s natural frequency, resonance effects can substantially increase the SPL. Table S2 summarizes tuning strategies for improving SP in specific frequency bands, including fixed single-resonator TASSs, discretely reconfigurable TASSs, and continuously tunable architectures from broader acoustic systems. Fixed and discretely reconfigurable resonators in TASSs provide limited enhancement or stepwise frequency switching (*20*, *39*–*42*). Nonetheless, to our knowledge, no TASS to date has achieved continuous resonance tuning through structural modulation. To explore feasible pathways, the third category introduces tunable acoustic systems that realize continuous adjustment via electrostatic, strain, magnetic, impedance, or geometric mechanisms. While these systems offer structural flexibility, their high voltage requirements and complex or rigid designs hinder integration into soft and wearable platforms (*43*–*52*). Developing TASSs with compliant architectures and simple, low-voltage continuous tuning remains a persistent challenge.

**Fig. 1.**
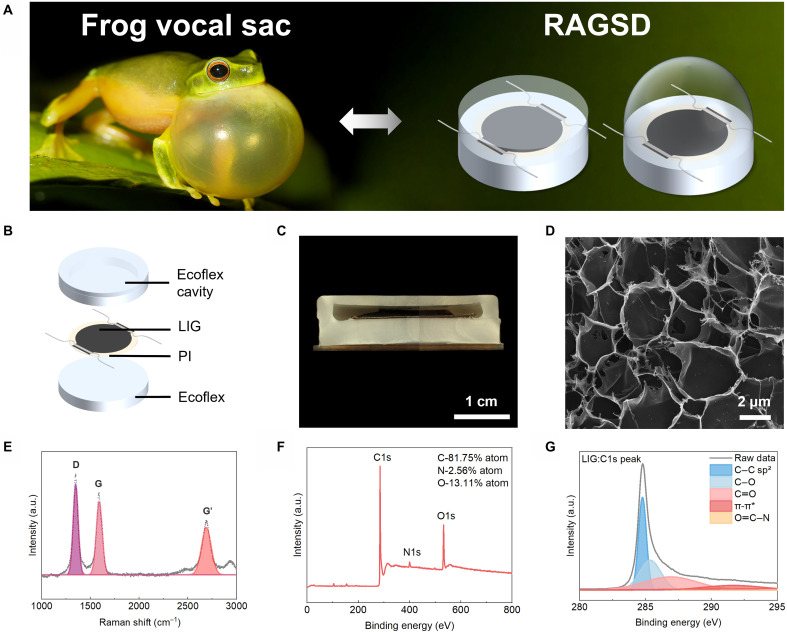
Schematic illustration of the structure and the morphology of RAGSD. (**A**) Comparative illustration of vocal sac inflation in frogs and gas inflation in the RAGSD (frog image https://www.istockphoto.com/photo/dainty-tree-frog-gm863310098-143125479, used under license). (**B**) Structure of RAGSD. (**C**) Cross-sectional optical microscopy image of the RAGSD. Scale bar, 1 cm. (**D**) SEM of LIG at high magnification. Scale bar, 2 μm. (**E**) The Raman spectrum obtained from the preparation of LIG tested with a laser power of 1.5 W. (**F**) The full spectrum XPS of LIG. (**G**) The C1s XPS of LIG. a.u., arbitrary units.

Male frogs use inflatable vocal sacs as natural acoustic resonators to amplify mating calls over long distances (Fig. 1A), exhibiting frequency-selective amplification well suited for soft and tunable systems (*53*, *54*). Inspired by this mechanism, a resonance-adjustable graphene sound device (RAGSD) is designed by integrating laser-induced graphene (LIG) with a deformable cavity. The RAGSD leverages the broadband emission of graphene and the tunable resonance of a soft cavity to enable frequency-selective enhancement across a wide acoustic range. The monolithic and stretchable architecture avoids rigid interfaces, and the compliance of the elastomer reduces fatigue under cyclic loading (*55*, *56*). The device achieves continuous resonance tuning from 922.12 to 1762.90 hertz (Hz) through modulation of the internal cavity volume (0 to 100 ml), covering a wide tuning range of 91.18%. To elucidate the tuning mechanism, a dynamic continuously tunable electro-mechano-acoustical (DCT-EMA) model is developed. Structural parameters, including diaphragm area, cavity volume, and membrane elasticity, are mapped to equivalent inductance and capacitance. The model predicts the resonance frequency as a function of cavity volume with high accuracy (coefficient of determination $R^{2}=0.990$ ) and retains robustness across variations in geometry and gas composition. These capabilities support two representative applications. In emission mode, an enhancement of 21.70 dB in SPL is observed at resonance, corresponding to a 12.19-fold increase in acoustic intensity compared to a nonencapsulated LIG membrane without external mechanical actuation, with an additional 3.64 dB improvement achieved when helium is used as the inflation gas. Frequency-selective enhancement was demonstrated by driving the RAGSD with vocal signals, enabling controllable amplification across the vocal frequency range and showing potential for personalized vocal assistance. Beyond enhancement, an emission-side and frequency-selective masking use case is further demonstrated enabled by continuous resonance tuning, where alignment to target sub-bands strengthens masking, thereby complementing the tunable enhancement with a tunable suppression pathway. In sensing mode, weak pathological heart sounds are selectively amplified by the tunable structure, and the piezoresistive response of LIG enabled sensitive transduction via resistance variation, allowing the device to clearly detect pathological murmurs such as ventricular septal defects (VSD) without probe switching. Beyond laboratory validation, wearable clinical tests on healthy volunteers and patients were conducted, in which the RAGSD captured clear S1 and S2 and enhanced pathological murmurs including aortic regurgitation under realistic hospital conditions. By enabling continuous resonance tuning, the device strengthened diagnostically relevant high-frequency components. When integrated with AuscNet-H, the system achieved an accuracy of 99.375% across four clinical heart sound categories; in the inflated condition, the model showed no false negatives and fewer misclassifications among similar diseases than a commercial electronic stethoscope. These results highlight the translational potential of the RAGSD, combining frequency-selective resonance tuning with data-driven classification for intelligent and wearable auscultation.

In summary, grounded in bio-inspired design, this work resolves the low-frequency bottleneck of TASSs, integrates a soft wearable emission sensing architecture, extends the DCT-EMA framework, and translates to unique applications in personalized phonation and auscultation; together with high-accuracy algorithms, it delivers a systems-level integrative innovation.

## RESULTS

### Structure and characterization of RAGSD

In this design, LIG, serving as the material for sound emission and sensing, is encapsulated within Ecoflex 00-30. The LIG and polyimide (PI) substrate are adhered to a bottom Ecoflex layer (Fig. 1B). To prevent direct contact with the LIG surface, a 3-mm gap is reserved between the bottom and the thinner upper Ecoflex membranes. The Ecoflex membrane on the upper surface of the RAGSD has a thickness of 0.5 mm, which is much thinner compared to the 3-mm side walls and the 4-mm base layer. Therefore, when air is injected into the device, the upper membrane expands upward, forming a hemispherical dome-shaped deformation. This membrane acts as the primary resonant element and serves as the resonator for the device. The LIG is fabricated under an optimized laser power of 2.34 W (note S1 and figs. S1 and S2). Figure 1C shows a cross-sectional view of RAGSD under an optical microscope, revealing its internal structure. Because of limitations in the field of view, the image is a composite of three separate images taken at different positions. As shown in Fig. 1D, the LIG film exhibits a porous and foam-like structure, which is attributed to the rapid release of gaseous by-products. This porous structure of graphene increases the specific surface area, thereby enhancing the interaction efficiency between sound waves and the material interface, ultimately improving its acoustic performance (*57*). The Raman spectrum exhibits three distinct peaks of LIG: the D peak at 1350 cm^−1^, corresponding to defects or distorted sp^2^ carbon bonds, the G peak at 1590 cm^−1^, representing the first-order allowed vibration mode, and the G′ peak at 2700 cm^−1^, indicative of interlayer vibration modes in graphene (Fig. 1E). Figure 1 (F and G) displays the full-spectrum and high-resolution C1s x-ray photoelectron spectroscopy (XPS) of LIG, respectively. The full-spectrum XPS further confirms the presence of nitrogen (N) and oxygen (O) doping in the LIG sample. The C1s XPS spectrum demonstrates that C─C bonds dominate in the LIG, while the C─O, C═O, O═C─N, and π-π* peaks from the PI are notably reduced, indicating that the LIG primarily consists of sp^2^ carbon, as shown in Fig. 1G (*58*).

### Sound source performance of RAGSD

**Fig. 2.**
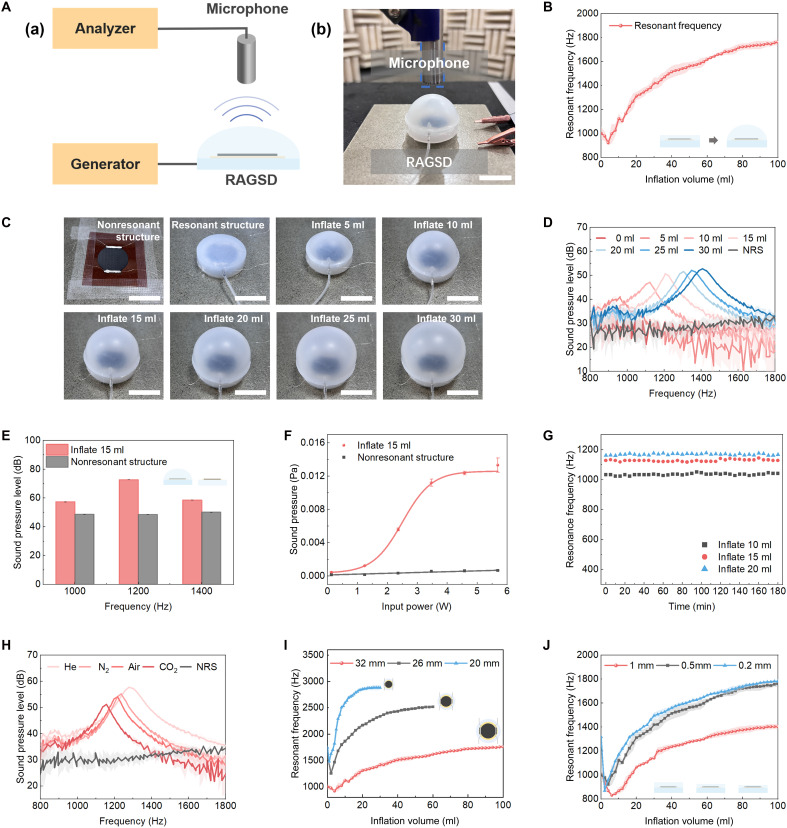
Sound emitting performance of RAGSD. (**A**) The generation and analysis system of RAGSD. (a) Schematic of RAGSD generation and analysis. (b) Photograph of RAGSD testing in an anechoic chamber, with the scale bar of 2 cm. (**B**) The relationship between the resonant frequency of RAGSD and the inflation volume. (**C**) Testing scenarios of RAGSD without and with resonant structures at inflation volumes of 0 to 30 ml, with the scale bar of 2 cm. (**D**) SPL response in the frequency domain for RAGSD with and without resonant structures at inflation volumes of 0 to 30 ml, measured at a distance of 3 cm and an input power of 1.67 W. (**E**) SPL response of RAGSD without and with resonant structures at an inflation volume of 15 ml under 1, 1.2, and 1.4 kHz. (**F**) The relationship between SP and input power for RAGSD without and with resonant structures at an inflation volume of 15 ml, measured at a distance of 3 cm, and a test sound frequency of 1.2 kHz. (**G**) Long-term stability of the resonant frequency of RAGSD over 180 min of continuous operation. (**H**) Frequency response of RAGSD inflated to 15 ml with different gases. (**I**) The relationship between resonant frequency and inflation volume for RAGSD with membrane areas of 20/26/32 mm. Inset: schematic illustration of membrane area variations. (**J**) The relationship between resonant frequency and inflation volume for RAGSD with membrane thicknesses of 0.2/0.5/1 mm. Inset: schematic illustration of membrane thickness variations.

The sound emission characteristics of the RAGSD are evaluated using the setup shown in Fig. 2A. A sinusoidal ac signal is applied to the LIG layer, generating acoustic waves on the membrane surface. These waves are detected by a standard microphone and analyzed using a frequency spectrum analyzer. All measurements are conducted in an anechoic chamber to minimize acoustic interference. The relationship between resonant frequency and inflation volume is presented in Fig. 2B. Air is injected into the device from 0 to 100 ml in 2-ml increments while sweeping the excitation frequency across the range of 20 Hz to 20 kHz. The frequency corresponding to the peak SPL at each step is recorded. The RAGSD achieved continuous tuning from 922.12 to 1762.90 Hz, yielding a wide tunable range of 91.18%, demonstrating precise acoustic modulation capability. Figure 2C shows the top-view deformation states under inflation volumes ranging from 0 to 30 ml, for both nonresonant and resonant configurations. Corresponding SPL responses are shown in Fig. 2D (input power: 1.67 W; microphone distance: 3 cm). Compared with the nonresonant structure (NRS), the RAGSD with a resonant membrane markedly enhanced the SPL at specific frequencies. The 0- to 30-ml span is selected, because it covers the rapid frequency-volume regime and centers a representative operating point at 15 ml near ~1.2 kHz, enabling clear, like-for-like comparison within the sensitive range. For example, under 15-ml inflation at ~1.2 kHz, the SPL increased by 21.70 dB, corresponding to a 12.19-fold enhancement in SP over the nonencapsulated configuration. As shown in Fig. 2E, the enhancement persists across adjacent frequencies (1.0, 1.2, and 1.4 kHz), with the resonant configuration consistently yielding higher SPL, indicating pronounced frequency selectivity near the resonance region. Figure 2F confirms that this enhancement remains stable across varying input powers (0 to 5.69 W), indicating that the frequency selectivity is independent of input amplitude. The long-term stability of the RAGSD is validated through a 180-min inflation test (Fig. 2G), showing negligible frequency drift, indicating excellent sealing and stability, which is verified by a 7-day checking at 15 ml (fig. S3A). Further 100-cycle inflation-deflation tests (fig. S4A) demonstrate strong fatigue resistance. A representative volume of 15 ml, together with flanking conditions of 10 and 20 ml, was selected to capture typical operating states within the sensitive tuning range. Figure S5 shows that excessive inflation may cause damage, so 100 ml is chosen as the safe limit.

Beyond inflation volume control, additional strategies are used to tune the resonance frequency and enhance the SPL. Figure 2H presents the frequency response under different inflation gases at a fixed volume of 15 ml. Gases with higher molecular mass, such as carbon dioxide (CO_2_), shift the resonance frequency downward, while gases with lower molecular mass, such as helium (He), shift it upward. Among the tested gases, helium inflation produces the highest SPL, reaching 59.73 dB. This corresponds to a 3.64-dB increase compared to air, confirming the effectiveness of gas substitution for acoustic performance enhancement. Figure 2I and fig. S6 show that increasing the membrane area (diameters of 20, 26, and 32 mm) lowers the resonance frequency, with larger devices operating within the subkilohertz range. Similarly, Fig. 2J demonstrates that increasing the diaphragm thickness from 0.2 to 1.0 mm also reduces the resonance frequency in a consistent manner. Acoustic emission tests under varying environmental and perturbation conditions show that lower temperature yields higher sound pressure levels (fig. S7A), humidity has a minimal effect (fig. S8A), and the RAGSD responds promptly to mechanical poking and rapidly returns to steady operation, with an average full-recovery time of ~80 ms (fig. S9A).

### Sound sensing performance of RAGSD

**Fig. 3.**
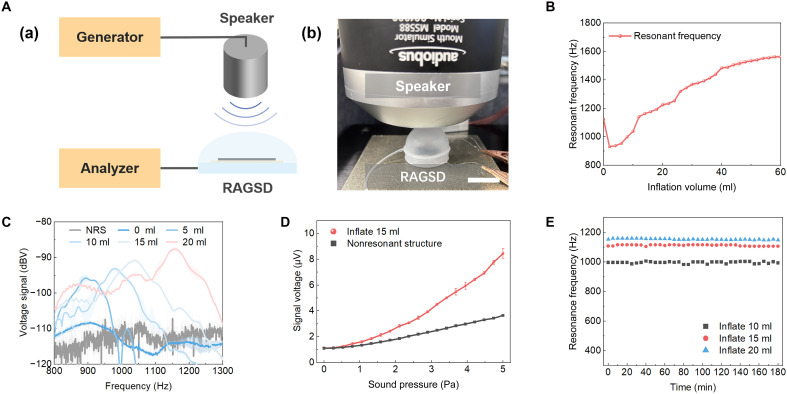
Sound sensing performance of RAGSD. (**A**) The analysis system of RAGSD. (a) Schematic of RAGSD generation and analysis. (b) Photograph of RAGSD testing in an anechoic chamber, with a scale bar of 2 cm. (**B**) The relationship between the resonant frequency of RAGSD and the inflation volume. (**C**) SPL response of RAGSD without resonant structure and with resonant structure inflated to 0/5/10/15/20 ml in the frequency domain, measured at a distance of 3 cm, with a standard speaker input voltage of 2 Vrms. (**D**) The relationship between the output voltage and input SP for RAGSD without and with the resonant structure, measured at an inflation volume of 15 ml, a distance of 3 cm, and a test sound frequency of 1.2 kHz. (**E**) Long-term stability of the resonant frequency of RAGSD over 180 min of continuous operation.

In addition to emission capabilities, the RAGSD can also be applied effectively as a sound sensor due to the piezoresistive properties of graphene. As illustrated in Fig. 3A, incident sound waves generated by a standard speaker are amplified by the resonant cavity of the RAGSD and transmitted to the LIG layer. The resulting mechanical vibrations induce changes in the resistance of the LIG, which are recorded and analyzed via a signal analyzer. All measurements are conducted in an anechoic chamber to minimize external acoustic interference. The relationship between the resonant frequency and inflation volume is shown in Fig. 3B. During the experiment, air is injected into the RAGSD in 2-ml increments from 0 to 60 ml while sweeping the input frequency from 100 Hz to 12 kHz. At each volume, the frequency corresponding to the peak SPL in the response curve is identified as the resonant frequency. Results demonstrate that the RAGSD exhibits continuously tunable resonance in sensing mode, with a tuning range comparable to that observed in emission mode. Figure 3C presents the frequency response curves of the RAGSD under inflation volumes of 0, 5, 10, 15, and 20 ml, with NRS included as the nonresonant control. In each case, a 2 root-mean-square voltage (Vrms) ac signal is applied to the speaker positioned 3 cm from the device. As observed, the resonant frequency shifts with increasing volume, confirming that the sensing behavior is volume dependent. Similar systematic tuning is also observed under direct contact conditions, with continuous tunability maintained; the additional effective mass introduced by contact causes a slight downshift in frequency, while the signal-to-noise ratio is improved (fig. S10). Figure 3D shows the sensor’s voltage response under acoustic excitation ranging from 0 to 5 Pa at 1.2 kHz (with 15-ml inflation). The response amplitude of the RAGSD is consistently higher than that of devices without resonant structures, validating its ability to selectively amplify acoustic signals at targeted frequencies. Notably, this amplification effect is independent of the absolute SPL, highlighting the device’s frequency selectivity. Long-term sensing stability is evaluated under continuous sound excitation for 180 min, with inflation volumes of 10, 15, and 20 ml (Fig. 3E), which is verified by a 7-day evaluation at 15 ml (fig. S3B). The resonant frequency is measured every 5 min. The RAGSD maintained consistent frequency characteristics over time, indicating reliable performance under extended operation. Further 100-cycle inflation-deflation tests (fig. S4B) confirm the device’s fatigue stability. Sensing tests under varied environmental and disturbance conditions indicate that lower temperature leads to higher sound pressure levels (fig. S7B), humidity has minimal impact (fig. S8B), and the RAGSD responds rapidly to mechanical perturbations and quickly returns to steady operation, with an average full-recovery time of ~80 ms (fig. S9B).

### DCT-EMA modeling for resonance tuning in RAGSD

**Fig. 4.**
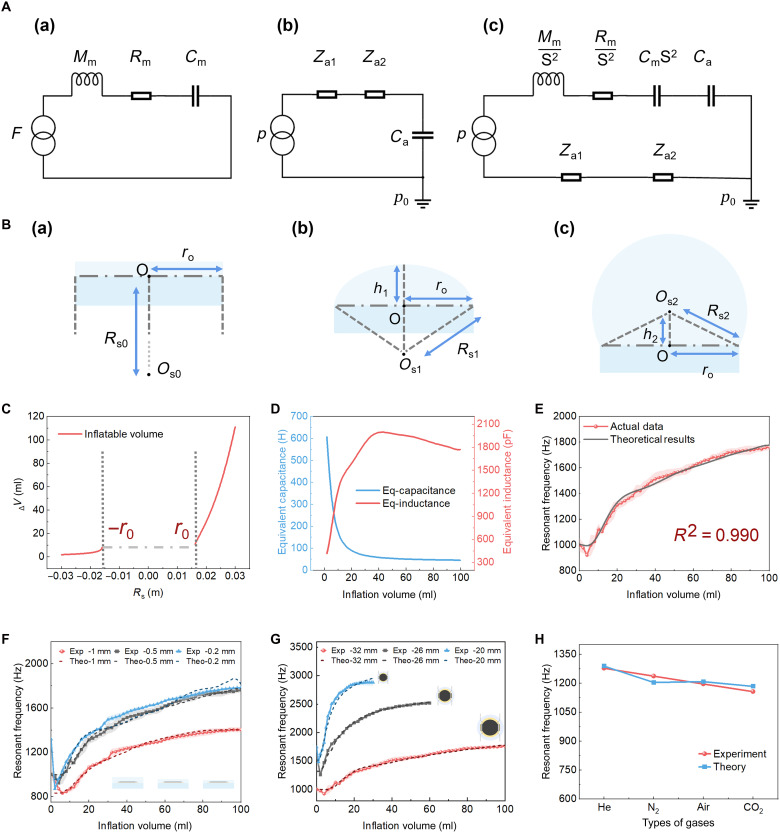
DCT-EMA modeling and experimental validation of tunable resonance in RAGSD. (**A**) Equivalent circuit representation of RAGSD: (a) mechanical-electrical analogy; (b) acoustic-electrical analogy; (c) fully coupled electro-mechano-acoustical circuit based on the DCT-EMA framework. (**B**) Schematic illustration of the gas inflation process in RAGSD: (a) initial state; (b) inflation with a small amount of gas; (c) inflation with a larger amount of gas. (**C**) Theoretical relationship between cavity volume and equivalent spherical diameter during inflation. (**D**) Model-derived relationship between equivalent inductance and capacitance and inflation volume. (**E**) Comparison between predicted and measured resonant frequency under inflation from 0 to 100 ml, with a coefficient of determination $R^{2}=0.990$ . (**F**) Model validation for resonant membranes with different thicknesses (0.2, 0.5, and 1 mm). (**G**) Model validation for devices with different diaphragm diameters (20, 26, and 32 mm). (**H**) Model validation under 15-ml inflation using different inflation gases: He, N_2_, air, and CO_2_, demonstrating robustness under varying gas properties.

A DCT-EMA model is developed to describe the resonance tuning behavior of RAGSD. This model extends classical EMA theory from rigid and static systems to soft, deformable acoustic structures (*59*–*61*). Mechanical, acoustic, and electrical domains are represented by equivalent inductive, capacitive, and resistive components (*62*), with circuit analogies illustrated in Fig. 4A (a to c). Full derivations are provided in note S2.

The model considers contributions from the deformable Ecoflex diaphragm, the gas-filled cavity, and the surrounding acoustic medium. The total equivalent inductance *L* consists of two parts: the mechanical mass *M*_m_ of the diaphragm scaled by the effective area *S*^2^ and the acoustic mass *M*_a_ contributed by the gas loading

$$L=M_{\text{total}}=\frac{M_{m}}{S^{2}}+M_{a}=\frac{\rho_{e}\pi r_{0}^{2}l_{0}}{S^{2}}+2\times 0.2705\times \frac{\rho_{g}}{r_{0}}$$ (1)

The total compliance *C* includes the elastic compliance of the diaphragm and the acoustic compliance of the gas cavity

$$C=C_{\text{total}}=C_{m}S^{2}+C_{a}=\frac{1}{k_{m}}S^{2}+\frac{V_{0}+∆V}{\gamma P_{g}}$$ (2)

The resonance frequency is then calculated as

$$f=\frac{1}{2\pi }\sqrt{\frac{1}{LC}}$$ (3)

Here, ρ_e_ is the density of Ecoflex 00-30, *r*_0_ and *l*_0_ are the diaphragm radius and thickness, ρ_g_ is the gas density, γ is the adiabatic index (table S3), *P*_g_ is the internal cavity pressure (fig. S11 and note S3), and *k*_m_ is the elastic modulus (fig. S12). This equation describes the closed-form relationship between resonant frequency and inflation volume. The quantitative values of the equivalent inductance *L* and capacitance *C* are determined on the basis of the geometric deformation of the diaphragm. Figure 4B illustrates the schematic stages of the inflation process, including the formation of the hemispherical dome. In Fig. 4C, an inverse solution is applied to establish the relationship between the inflation volume and the equivalent spherical radius, and the shaded interval from −0.016 to 0.016 m denotes a mathematically undefined region in the geometric abstraction. Figure 4D presents the derived relationship between the inflation volume and the calculated values of *L* and *C*, forming the basis for frequency prediction under varying structural conditions. As shown in Fig. 4E, the predicted frequency response closely matches experimental data across the range of 0 to 100 ml, yielding a coefficient of determination $R^{2}=0.990$ , which validates the model’s accuracy.

To further assess the model’s generality and robustness, a series of comparative experiments is conducted under varying structural and environmental conditions (Fig. 4, F to H). Figure 4F shows frequency tuning under different diaphragm thicknesses (0.2, 0.5, and 1 mm). Figure 4G evaluates predictions for different diaphragm diameters (20, 26, and 32 mm). Figure 4H is the model’s result under various inflation gases [He, nitrogen (N_2_), air, and CO_2_]. In all cases, the model maintains strong predictive performance with $R^{2}>0.970$ , demonstrating applicability across geometries, materials, and gas environments. The simulation results for SP distribution and frequency response of the RAGSD are consistent with the experimental trends (fig. S13). These results confirm that the DCT-EMA framework offers a generalized and scalable approach for modeling soft and tunable acoustic systems.

### Dual-function emission and sensing applications

**Fig. 5.**
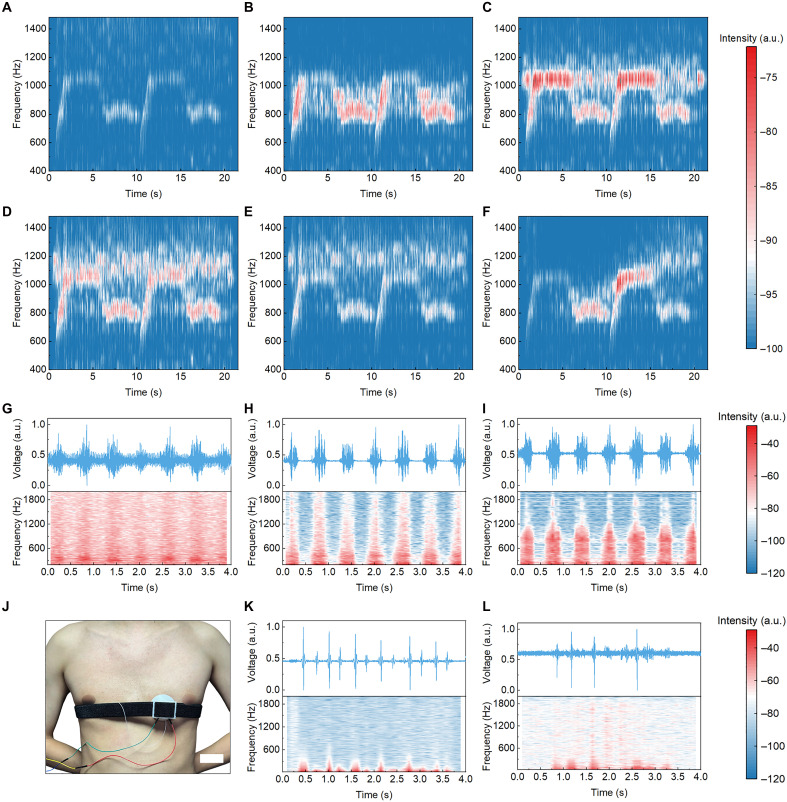
Dual-function acoustic capability of the RAGSD for emission and sensing. (**A** to **F**) Spectrograms of RAGSD acoustic emission driven by an ac-modulated audio waveform (chorus segment of *Opera No. 2*) under different inflation volumes, measured at a distance of 3 cm and an input power of 3.27 W: 0 ml (A), 5 ml (B), 10 ml (C), 15 ml (D), 20 ml (E), and continuous uniform inflation from 0 to 20 ml (F). (**G** to **I**) Normalized time-domain waveforms (top) and spectrograms (bottom) of heart sounds from a patient with VSD, recorded using an uninflated RAGSD (G), a RAGSD inflated to 15 ml (H), and a commercial electronic stethoscope (I). (**J**) Photograph of the wearable RAGSD stethoscope system mounted on a healthy subject, showing the encapsulated RAGSD probe. Scale bar, 5 cm. (**K**) Time-domain waveform (top) and corresponding time-frequency spectrogram (bottom) of heart sounds from a healthy subject. (**L**) Time-domain waveform (top) and corresponding time-frequency spectrogram (bottom) of heart sounds from a patient with aortic regurgitation.

The RAGSD can realize both acoustic emission and sensing, demonstrating its dual-function capability for diverse application scenarios. In terms of sound emission, its continuously tunable resonance frequency enables it to be applied as an auxiliary vocalization component. In this study, an ac-modulated audio waveform representing the chorus segment of *Opera No. 2* is input into the RAGSD, and the spectrogram of the original audio is shown in fig. S14A. Acoustic outputs are recorded using a standard microphone under inflation volumes of 0, 5, 10, 15, and 20 ml, as well as under continuous uniform inflation from 0 to 20 ml. The recorded signals are normalized and transformed into spectrograms (Fig. 5, A to F, and fig. S15). A dynamic demonstration of the acoustic modulation process is provided in movie S1. The spectrograms show that as the inflation volume increases, the resonant frequency of the RAGSD shifts progressively upward, accompanied by a redistribution of acoustic energy across the frequency spectrum. In addition, we implemented a frequency-selective masking demonstration on the emission side: by tuning the RAGSD peak to align with discrete high-frequency components of a dual tone multifrequency target, masking strengthened when aligned (fig. S16), thereby complementing the continuously tunable enhancement with a continuously tunable suppression pathway. These results confirm that the resonant characteristics of the device can be finely adjusted by modulating the internal chamber volume and provide a feasible approach to achieving customized acoustic output.

In terms of sound sensing, the RAGSD addresses the limitations of traditional stethoscopes, which typically rely on fixed geometries and a single inherent resonance mode, limiting their ability to capture a broad range of heart sound frequencies. Clinicians often need to switch different stethoscope probes, such as flat, bell-shaped, or diaphragm types, to meet diagnostic requirements. In contrast, the RAGSD enables real-time resonance tuning via controlled inflation, allowing for frequency-selective enhancement of specific acoustic components without mechanical switching. Under normal conditions, the acoustic energy of healthy heart sounds is primarily concentrated below 300 Hz (fig. S14B). However, in the presence of structural heart diseases such as VSD, aortic stenosis (AS), mitral regurgitation (MR), and mitral valve prolapse (MVP), turbulent blood flow and pressure gradients can generate abnormal high-frequency components. These pathological sounds often exceed 1 kHz and may reach beyond 2 kHz (fig. S14, C to F). Notably, such high-frequency murmurs are typically weak during the early stages of disease progression and prone to being missed by conventional stethoscopes, potentially leading to false-negative diagnoses and delayed clinical intervention. Figure 5 (G to I) presents normalized time-domain waveforms and corresponding spectrograms of heart sounds from a patient diagnosed with VSD, recorded using three sensing configurations: Fig. 5G shows an uninflated RAGSD, Fig. 5H shows a RAGSD inflated to 15 ml, and Fig. 5I shows a commercial electronic stethoscope, which includes built-in signal conditioning modules such as a preamplifier and filtering circuit (fig. S17 and table S4). The commercial stethoscope exhibits a relatively flat frequency response and performs well in the lower-frequency range (<1 kHz) but shows limited sensitivity to high-frequency murmurs associated with pathological sites (fig. S18). The uninflated RAGSD provides moderate signal enhancement with limited frequency selectivity. In contrast, the RAGSD inflated to 15 ml shows clear amplification of murmur components around 1.2 kHz. To further verify practical applicability, a portable wearable intelligent stethoscope system is developed. Figure S19 shows the complete system, consisting of a stethoscope head module [RAGSD with dedicated encapsulation (fig. S20), elastic strap, air injector, and seals], a compact acquisition circuit, and a host computer for real-time preprocessing and display. Within this system, a compact 5-kHz acquisition circuit and a PCB-controlled micropump with flexible tubing are implemented. Figure S21 shows the hardware, and movie S2 shows inflation and deflation cycles. To ensure stable coupling, the RAGSD was enclosed in the headpiece and secured by an elastic strap for continuous monitoring. In healthy subject tests (Fig. 5J), clear S1 and S2 heart sounds were obtained, with corresponding time and time-frequency analyses shown in Fig. 5K. Resonance tuning selectively enhanced diagnostically relevant frequency bands, demonstrating feasibility and stability in a wearable context. Movie S3 shows the wearable use of RAGSD in a hospital environment, where the device records cardiac sounds for 1 min from a patient with aortic regurgitation. In patient tests (fig. S22A), recordings from the aortic second intercostal and mitral areas yielded the time- and time-frequency results in Fig. 5L and fig. S22B. Despite a slight reduction in the signal-to-noise ratio in the patient data compared to the healthy subject, which resulted from subject movement in the supine position and a looser strap fit for patient comfort and safety, the recorded signals still exhibited stronger spectral energy above 1 kHz. This finding is consistent with the acoustic characteristics of regurgitant murmurs and demonstrates that the wearable RAGSD effectively captures clinically relevant pathological heart sounds even under realistic measurement conditions. To assess robustness under ambient interference, movies S4 (quiet) and S5 (noisy) show real-time on-skin recordings from the same subject and mounting; fig. S23 presents the paired analysis, where the waveforms are consistent and the signal-to-noise ratio decreases only marginally in noise (~0.22 dB), indicating strong environmental robustness.

To ensure dual-function use is sustainable, note S4 links application to durability. Scenarios are mapped to lifetime: Operation near 30 ml corresponds to ~10^4^ cycles, whereas rare 100 ml use corresponds to ~10^3^ cycles. Cap analysis gives λ ≈ 2.235 (30 ml) and λ ≈ 3.47 (100 ml), guiding uniaxial cycling at 150% (10,000 loops; fig. S24A) and 300% (1000 loops; fig. S24B). Loops remain consistent with less mechanical degradation; the ~108-kPa stress at maximum inflation is far below the ~488.85-kPa reference (fig. S24C). Together with device stability tests, this supports long-term use.

These results demonstrate that the tunable resonance structure of the RAGSD enables frequency-specific signal amplification, improving the detection of weak, high-frequency pathological sounds and enhancing the effectiveness of cardiac auscultation. By integrating portable electronics and a wearable configuration, the system not only confirms feasibility under controlled laboratory conditions but also shows tangible potential for real-world clinical applications, providing a solid foundation for the translation of intelligent auscultation into practice.

### Intelligent stethoscope based on AuscNet-H deep learning algorithm

**Fig. 6.**
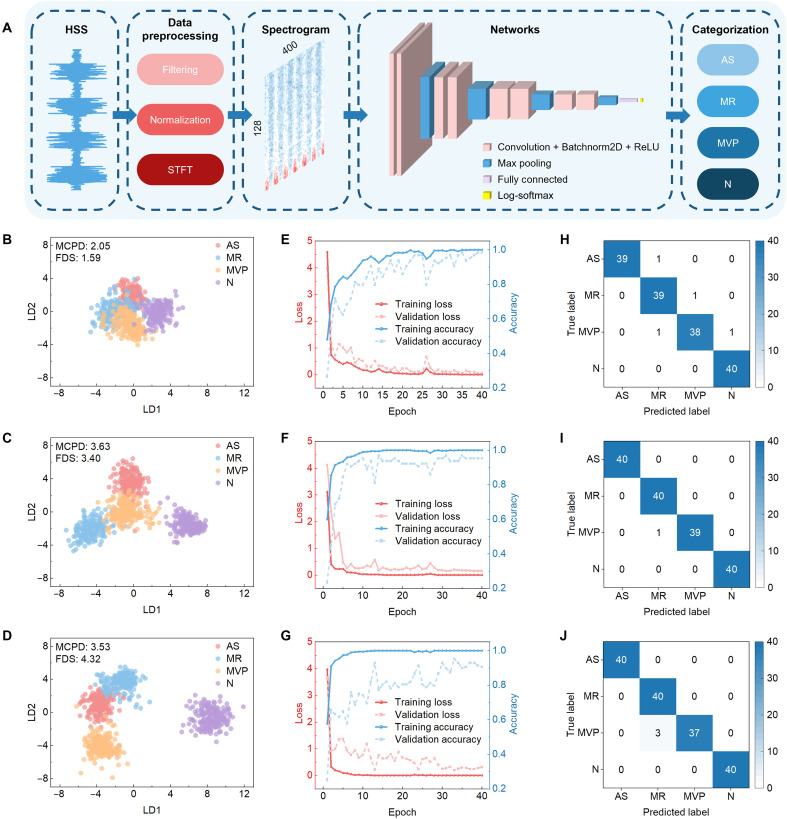
Evaluation of heart sound classification performance using AuscNet-H. (**A**) Workflow of signal preprocessing and classification. Heart sound signal (HSS) is filtered, normalized, converted into 128 × 400 spectrograms via STFT, and input into AuscNet-H. (**B** to **D**) LDA projections of extracted features: 0-ml dataset (MCPD = 2.05, FDS = 1.59) (B), 15-ml dataset (MCPD = 3.63, FDS = 3.40) (C), and commercial electronic stethoscope dataset (MCPD = 3.53, FDS = 4.32) (D). (**E** to **G**) Training and validation curves over 40 epochs: 0-ml dataset (E), 15-ml dataset (F), and commercial electronic stethoscope dataset (G). (**H** to **J**) Confusion matrices corresponding to the three datasets: 0-ml dataset with an overall accuracy of 97.5% (H), 15-ml dataset with an overall accuracy of 99.375% (I), and commercial electronic stethoscope dataset with an overall accuracy of 98.125% (J).

Deep learning has shown strong capabilities in identifying pathological acoustic signatures (*63*–*65*). In this study, AuscNet-H, a deep learning heart sound classification algorithm, is used to evaluate the impact of RAGSD-enabled resonance tuning on intelligent auscultation. To assess diagnostic enhancement via RAGSD, four heart sound categories [AS, MR, MVP, and normal (N)] are selected from a public dataset (200 samples per class) (*66*). All audio clips are extended to 4 s and normalized in loudness (fig. S25). The signals were played through a standard speaker and recorded by RAGSD under uninflated (0 ml) and inflated (15 ml) conditions, as well as by a commercial electronic stethoscope, generating three datasets of 800 samples each. The voltage versus time signals are processed with a fifth-order Butterworth bandpass filter, *z*-score normalized, and transformed into 128 × 400 spectrograms using short-time Fourier transform (STFT) (Fig. 6A) (*67*). To ensure that the test set more closely reflects the complex acoustic environment of clinical auscultation and better simulates real-world conditions, preprocessing was applied only to the training set but not to the test set (*68*, *69*). AuscNet-H consists of four convolutional blocks (each with 2×Conv2D, BatchNorm2D, ReLU, and MaxPooling), followed by global max pooling, a 512-unit fully connected layer, and a log-softmax output. The datasets are separately divided into 80% training and 20% test, with 10% of training samples used for validation.

The performance of RAGSD integrated with AuscNet-H is evaluated through feature extraction and classification analysis. Figure 6A illustrates the workflow of signal preprocessing and classification, with three datasets compared: RAGSD at 0- and 15-ml inflation and a commercial electronic stethoscope. Because the model consumes a standardized time-frequency representation, the pipeline is front-end agnostic and accepts digital audio from different stethoscopes. In the LDA projections, the 0-ml dataset yielded Minimum Class Pair Distance (MCPD) = 2.05 and Fisher Discriminant Score (FDS) = 1.59 (Fig. 6B); both scores increased for the 15-ml dataset (MCPD = 3.63, FDS = 3.40; Fig. 6C), showing that inflation at the corresponding volume selectively amplifies diagnostically relevant frequency bands, consistent with the resonance-tuning mechanism of the device. The commercial stethoscope achieved a higher overall discriminant score (MCPD = 3.53, FDS = 4.32; Fig. 6D), mainly owing to its superior low-frequency performance enabling stronger separation of normal and abnormal heart sounds; however, its MCPD was lower than that of the 15-ml dataset, indicating weaker discrimination among similar pathological conditions (*70*). The training and validation curves further support this trend (Fig. 6, E to G). Over 40 training epochs, the 0-ml dataset converged more slowly with larger fluctuations; the 15-ml dataset converged faster and more stably; the commercial stethoscope converged slightly faster in training but showed decreased validation accuracy, suggesting limited robustness in disease-to-disease separation. Additional confusion matrix analysis at various training epochs also supports these findings (fig. S26). Final confusion matrices (Fig. 6, H to J) showed high accuracies for all three datasets: 97.5% (0 ml), 99.375% (15 ml), and 98.125% (stethoscope). Notably, the 15-ml dataset yielded no false negatives across four clinical categories, avoiding missed diagnoses, and exhibited fewer misclassifications among similar diseases, thereby reducing misdiagnosis compared to the stethoscope. The device without inflation still retained reliable feature extraction capability while confirming the robustness of the AuscNet-H algorithm under varying data conditions. To further evaluate generalization to new human subjects in complex environments, the on-body tests are conducted in nonanechoic settings with posture changes (movie S6) and adopted two-class inference at test time, collapsing AS, MR, and MVP into abnormal and retaining N as normal. The confusion matrices for a patient in supine and seated postures and for three healthy subjects in seated, standing, and slow-walking postures show 100% correct classifications (fig. S27), indicating reliable performance under wearable conditions across complex environments and varying motion states.

In summary, the selective amplification enabled by the inflated device (physical front-end) and the efficient feature extraction of AuscNet-H (algorithmic back-end) act synergistically to substantially enhance intelligent heart sound classification, providing dual assurance for future wearable and clinical applications. Table S5 presents the pseudocode for the implementation of AuscNet-H.

## DISCUSSION

Inspired by the inflatable vocal sacs of frogs, this study presents a deformable acoustic device capable of thermoacoustic emission and piezoresistive sensing. By adjusting internal cavity volume, RAGSD enables continuous resonance tuning over 922.12 to 1762.90 Hz (91.18%), effectively addressing the limited low-frequency SPL output of conventional thermoacoustic systems. At resonance, the SPL is enhanced by 21.70 dB, equivalent to a 12.19-fold increase in SP, supporting frequency-selective control in both emission and sensing.

To explain this tunability, a DCT-EMA model is developed. The model maps structural and material parameters, such as diaphragm geometry, membrane elasticity, cavity volume, gas properties, and deformation area, into equivalent circuit elements. Unlike previous EMA models limited to rigid, static systems, DCT-EMA expands the theoretical framework to accommodate soft, reconfigurable acoustic structures and shows strong agreement with experimental results across the tuning range ( $R^{2}=0.990$).

These capabilities can realize two experimentally validated applications. In emission mode, resonance tuning enables selective amplification of user-defined acoustic content, such as low-frequency speech, tonal prompts, or assistive output, supporting personalized sound enhancement. In addition, an emission-side frequency-selective masking demonstration is implemented enabled by continuous resonance tuning, where alignment to target sub-bands strengthens masking, providing a tunable suppression pathway complementary to enhancement. In sensing mode, the resonant structure selectively amplifies weak pathological features, such as high-frequency murmurs in VSD heart sounds, helping clinicians better detect subtle acoustic signals. Unlike traditional stethoscopes with fixed-shaped heads and limited frequency coverage, RAGSD eliminates the need to switch between chest pieces, streamlining operation and improving diagnostic accessibility. Beyond laboratory validation, we conducted wearable clinical tests on healthy volunteers and patients, in which the device captured clear S1, S2, and amplified pathological murmurs including aortic regurgitation under hospital conditions. Continuous resonance tuning strengthened diagnostically relevant high-frequency bands, further illustrating the advantage of frequency-selective enhancement in realistic measurements. Despite these advantages, the prototype still needs an external gas source and power, and its inflated dome is less conformal; future versions will integrate miniaturized gas control and portable power and adopt more conformal packaging to improve portability and comfort.

When integrated with AuscNet-H, the system achieved an accuracy of up to 99.375% across four clinical heart sound categories. In the inflated condition, no false negatives were observed, and misclassifications among similar diseases were fewer than with the commercial electronic stethoscope, consistent with the device-level comparison that inflation mitigated missed detections.

In summary, RAGSD demonstrates a soft and tunable acoustic platform that combines mechanical adaptability with intelligent signal interpretation. The robust theoretical basis and experimentally confirmed functionality position it as a promising solution for smart auscultation, flexible biomedical monitoring, and wearable acoustic diagnostics. The synergy between continuously tunable front-end resonance and data-driven classification provides dual assurance for real-world clinical auscultation, supporting translation toward wearable diagnostic systems.

## MATERIALS AND METHODS

### Fabrication of RAGSD

A 0.25-mm-thick PI film (Shenzhen Yingshida Plastic Materials Co. Ltd.) is positioned under a laser engraving machine (K6-pro, Shanghai Diaotu Industrial Co. Ltd.). A 450-nm laser with a maximum power of 3 W is used to directly write a porous LIG pattern with a 2-cm diameter on the PI surface. Electrodes are formed by coating conductive silver paint on both ends of the LIG region, connecting 0.1-mm-diameter silver wires as leads. A three-dimensional printer (Formlabs Inc.) is used to produce the encapsulation structure of RAGSD, consisting of upper and lower parts. Ecoflex 00-30 silicone (Smooth-On Inc., A:B = 1:1) is poured evenly into the mold, and the structure is demolded after curing for 1 hour at ambient temperature. The PI substrate with LIG is then attached to the lower part of the Ecoflex structure using silicone adhesive (DOWSIL 3140 RTV Coating, Dow Inc.). The upper and lower Ecoflex parts are bonded, with a thin layer of Ecoflex 00-30 applied to seal the LIG within the Ecoflex cavity, allowing the silver wires to extend through the joining area. Small holes are punctured around the upper Ecoflex structure, where a small segment of tubing is adhered to enable device inflation. The structure is left to cure for an additional hour at room temperature, after which excess Ecoflex around the edges is trimmed to finalize the RAGSD fabrication.

### Characterization

The surface morphology of the LIG is observed using an XL G2 desktop scanning electron microscope (Phenom Inc.). Raman spectroscopy is performed with a 532-nm laser (Zolix Instruments Co. Ltd.). XPS full-spectrum and high-resolution narrow-scan analyses are conducted using an x-ray photoelectron spectrometer (Thermo Fisher Scientific Inc.).

### Acoustic performance testing

The acoustic testing platform (Dongguan Aopuxin Audio Technology Co. Ltd.) comprised a standard microphone, a standard speaker, a power amplifier, and a dynamic signal analyzer. The dynamic signal analyzer generated signals from 20 Hz to 20 kHz and analyzed the acquired signals. A ^1^/_2_-inch (1.27 cm) free-field microphone is used to measure the sound produced by RAGSD, while a standard speaker with a frequency range of 100 Hz to 12 kHz is used to produce sound for the RAGSD’s sensing testing. The testing platform is enclosed with sound-absorbing foam to ensure a quiet environment. Figure S28 shows the sensitivity spectrum of the standard microphone and the frequency response of the standard speaker in the anechoic chamber. Both devices exhibit flat responses within the range of 100 Hz to 10 kHz, demonstrating the standardized and controlled acoustic testing conditions.

### Mechanical performance evaluation

Ecoflex 00-30 specimens were analyzed on a single-column microcomputer-controlled universal testing machine (XLD-100E, Guangzhou Jingkong Mechanical Testing Co. Ltd.). The uniaxial compression test was conducted under displacement control at a strain rate of 2 mm/min in a single-speed loading mode. In the cyclic tensile test and the maximum strain tensile test within the measurable range of the universal testing machine (UTM), the Ecoflex 00-30 specimens were prepared into C-shape dumbbell specimens (ASTM D412-C geometry) with a thickness of 3 mm and tested under displacement control at a strain rate of 500 mm/min. Before formal tensile testing, specimens were preloaded via the UTM grips to remove slack.

### Statements

All human trials involved in this article have passed the scientific review of The Seventh Affiliated Hospital of Sun Yat-sen University (Shenzhen) Institutional Review Board. The number of the study is: Medical Ethics of KY-2024-037-02.

## Supplementary Materials

### The PDF file includes:

Supplementary Materials and Methods Figs. S1 to S28 Tables S1 to S7 Legends for movies S1 to S6 References
