# Supplementary material for: Frog vocal sacs-inspired soft acoustic system with continuously tunable resonance for sound emission and stethoscopic sensing
Source: Sci Adv. 2025 Dec 17;11(51):eadz5930. doi: 10.1126/sciadv.adz5930 (PMC12710722; doi:10.1126/sciadv.adz5930)
Supplement: Supplementary file 1 — Supplementary Materials and Methods Figs. S1 to S28 Tables S1 to S7 Legends for movies S1 to S6 References [file sciadv.adz5930_sm.pdf]

Science Advances
AAAS

Supplementary Materials for

Frog vocal sacs-inspired soft acoustic system with continuously tunable resonance for sound emission and stethoscopic sensing

Chuting Liu *et al.*

Corresponding author: Yancong Qiao, qiaoyc3@mail.sysu.edu.cn; Jianhua Zhou, zhoujh33@mail.sysu.edu.cn; Tian-Ling Ren, rentl@tsinghua.edu.cn

Sci. Adv. **11**, eadz5930 (2025)
DOI: 10.1126/sciadv.adz5930

The PDF file includes:

Supplementary Materials and Methods
Figs. S1 to S28
Tables S1 to S7
Legends for movies S1 to S6
References

Other Supplementary Material for this manuscript includes the following:

Movies S1 to S6

Supplementary Materials and Methods

1. Justification of Laser Power Selection for LIG Fabrication.

To determine a suitable laser power for fabricating laser-induced graphene (LIG) with stable acoustic and electrical performance, we systematically evaluated the influence of laser power on key physical parameters of the LIG films. The tested laser power range was 1.2 W to 3.0 W, with the scan speed and focal conditions kept constant to isolate the effect of power. As shown in Fig. S1(B to D), the measured sound pressure levels (SPL) at 1 kHz and 5 kHz, as well as the sheet resistance of the LIG films, exhibit a correlated trend across power settings.

Specifically, when the laser power is set around 2.34 W, the LIG exhibits relatively high SPL and relatively low electrical resistance, indicating that the graphene structure formed under this condition supports efficient Joule heating and thermoacoustic energy conversion. SEM images in Fig. S2 confirm that this power level yields a porous and uniform surface morphology that enhances thermal exchange with the surrounding air. In contrast, at lower power levels (<1.2 W), the reduced thermal input leads to insufficient carbonization, resulting in elevated resistance and limited acoustic output. At higher powers (>2.7 W), local overheating induces surface ablation, pore collapse, or debris accumulation, all of which degrade the acoustic performance and structural uniformity.

Considering both the acoustic performance and fabrication stability, 2.34 W is selected as the standard laser power for LIG processing in this work. This setting ensures reproducible film quality, consistent thermoacoustic behavior, and robust device integration throughout all subsequent experiments.

2. DCT-EMA Modeling Derivation.

To quantitatively model the tunable resonance behavior of the RAGSD, a DCT-EMA model is developed. This model extends classical EMA analogies, which were originally developed for rigid and static systems, to soft and geometrically deformable acoustic platforms. In this framework, mechanical, acoustic, and electrical subsystems are unified through equivalent inductive, capacitive, and resistive circuit components (*71-73*). The focus of this derivation is to establish the relationship between resonant frequency and gas inflation volume by determining the expressions and numerical values for the equivalent inductance L and capacitance C of the system. For clarity, the equivalent circuit diagrams and deformation schematics used in this theoretical derivation refer to Fig. 4 in the main text. Since both the emission and sensing functions are realized within the same device structure, with consistent membrane geometry, cavity volume, boundary conditions, and material composition, the system can be considered to exhibit quasi-acoustic reciprocity (*74*). This assumption supports the use of a unified theoretical model for both modes. Although the structure and modeling approach are the same, minor differences in energy coupling and acoustic loading may lead to small variations in resonance behavior. These differences do not affect the primary resonance mechanism, and the unified model remains applicable. The DCT-EMA model in this work describes the structure-driven acoustic and mechanical response together with its equivalent electrical representation. The electrical and thermal excitation is treated as a prescribed source term, and the model is not intended to provide a complete transduction chain from electrical input to acoustic output. A comprehensive summary of all variables and parameters used in the theoretical model is provided in Table S6.

2.1 Mechanical Subsystem

The diaphragm is modeled as a mechanical oscillator characterized by its mass M_m, damping resistance R_m, and elastic compliance $C_m=1/k_m$. The elastic coefficient k_m is obtained from uniaxial compression tests and determined by fitting the linear slope of the stress–strain curve (Fig. S12) (*75*).

$$Z_m = R_m + j\left(\omega M_m - \frac{1}{\omega C_m}\right) \tag{S1}$$

The diaphragm mass is defined as:

$$M_m = \rho_e \pi r_0^2 l_0 \tag{S2}$$

2.2 Acoustic Subsystem

The acoustic load includes two radiative impedance components (Z_{a1}, Z_{a2}) and a cavity compliance term C_a. The total acoustic impedance is expressed as:

$$Z_a = Z_{a1} + Z_{a2} + \frac{1}{j\omega C_a} \tag{S3}$$

When $kr_0<0.5$, the radiation impedance for a circular diaphragm in free space is approximated by:

$$Z_{a1} = Z_{a2} = R_a + M_a \quad (S4)$$

$$M_a = 0.2705\frac{\rho_g}{r_0} \quad (S5)$$

Here, ρ_g is the gas density and r_0 is the diaphragm radius.

The acoustic compliance of the sealed chamber is given by (*76, 77*):

$$C_a = \frac{V}{\gamma P_g} = \frac{V_0 + \Delta V}{\gamma P_g} \quad (S6)$$

where γ denotes the adiabatic index of the gas inside the cavity, P_g is the gas pressure in the cavity, V_0 is the initial cavity volume, and ΔV is the volume of injected gas. The adiabatic index γ depends on gas type and temperature (Table S3), enabling resonance frequency tuning of the RAGSD by inflating with different gases (Fig. 4H). The gas pressure P_g is measured using a barometer, and its relationship with gas volume is established via polynomial fitting (Fig. S11, Note S2).

2.3 Geometric Inflation Modeling

To quantitatively describe the deformation of the flexible diaphragm during inflation, a geometric abstraction model is developed, which captures the evolution of the cavity's shape and volume. Unlike rigid chambers with fixed geometries, the RAGSD features a highly deformable top membrane that undergoes large, nonlinear deformation under air pressure, transitioning from a planar state to a spherical-cap-like configuration. Accurate modeling of this transformation is crucial for evaluating the effective acoustic area and, consequently, the equivalent electrical parameters in the DCT-EMA framework.

The geometric transformation is defined using an equivalent spherical radius R_s, with the initial plane of the device taken as the horizontal reference. The deformation process is divided into two mathematically tractable stages:

Stage 1: When the injected gas volume is relatively small, the center of the spherical cap lies below the initial membrane plane, corresponding to a negative equivalent spherical radius ($R_{s1}<0$). The inflation volume ΔV and cap height h_1 satisfy:

$$\Delta V = \frac{1}{6}\pi h_1(3r_0^2 + h_1^2) \quad (S7)$$

$$h_1 = -R_1 - \sqrt{R_{s1}^2 - r_0^2} \quad (S8)$$

Stage 2: With further inflation, the center of curvature crosses above the membrane plane,

corresponding to a positive spherical radius (R_{s2}>r_0). The expression becomes:

$$\Delta V = \frac{4}{3}\pi R_{s2}^3 - \frac{1}{6}\pi h_2(3r_0^2 + h_2^2) \tag{S9}$$

$$h_2 = R_{s2} - \sqrt{R_{s2}^2 - r_0^2} \tag{S10}$$

The transition zone $-r_0<R_s<r_0$ corresponds to a mathematically undefined region where the center of curvature lies near the diaphragm plane and is excluded from the fitting domain.

This model formulates a mathematical mapping between the injected gas volume and the diaphragm's geometric deformation. Based on this geometric framework, an inverse solution is derived to relate gas volume to the equivalent spherical radius (Fig. 4C), which is subsequently used to calculate the variation of effective radiating area S with inflation volume. The gray dashed box in Fig. 4C marks a mathematically undefined domain from -0.016 m to 0.016 m that also lacks a physically meaningful deformation, and it is excluded from fitting. This provides a theoretical basis for modeling the volume-dependent inductance and capacitance in the DCT-EMA framework.

2.4 Coupled Circuit and Resonance Frequency

By dimensional mapping between mechanical and acoustic domains through the transformer S:1, the coupled impedance becomes:

$$Z_{\text{total}} = R_{\text{total}} + j\left(\omega M_{total} - \frac{1}{j\omega C_{total}}\right) \tag{S11}$$

with total acoustic mass and compliance:

$$M_{total} = \frac{M_m}{S^2} + M_a = \frac{\rho_e \pi r_0^2 l_0}{S^2} + 2 * 0.2705 * \frac{\rho_g}{r_0} \tag{S12}$$

$$C_{total} = C_m S^2 + C_a = \frac{1}{k_m} S^2 + \frac{V_0 + \Delta V}{\gamma P_g} \tag{S13}$$

Finally, the resonant frequency is expressed as:

$$f = \frac{1}{2\pi}\sqrt{\frac{1}{M_{total}C_{total}}} \tag{S14}$$

This expression links frequency tuning directly to geometric and material parameters and has been shown to accurately match experimental data with a coefficient of determination (Fig. 4, E to H).

3. Internal Pressure Measurement and Polynomial Fitting for the Resonant Cavity.

To characterize the inflation behavior of the RAGSD, a series of internal pressure–volume measurements are conducted under different structural and gas conditions. The experimental setup is shown in Fig. S11A, where a precision syringe is used to inject gas into the sealed cavity, and the resulting pressure is recorded using a digital barometer.

Fig. S11B presents the pressure-volume relationship for air inflation, along with a polynomial fitting curve. The experimental data are fitted using a ninth-order polynomial function (*78*), expressed as:

$$P(V) = a_0 + a_1V + a_2V^2 + \cdots + a_9V^9 \tag{S15}$$

where P denotes internal pressure and V is the inflation volume. The fitting yields a high coefficient of determination ($R^2 = 0.999$).

To evaluate the impact of structural parameters, measurements are repeated for membranes of different thicknesses (Fig. S11, C and D) and diameters (Fig. S11, E and F). Thicker membranes result in higher internal pressures under the same volume due to increased mechanical stiffness.

Additionally, the effect of inflation gas is investigated. Air is replaced with helium, nitrogen, or carbon dioxide, and the corresponding pressure-volume curves are shown in Fig. S11 (G to I). These data enable accurate determination of gas pressure P_g under different conditions, which is essential for the DCT-EMA model.

The fitting coefficients a_0 to a_9 and corresponding coefficients of determination (R^2) for each condition are summarized in Table S7. These parameters are used for pressure prediction in the analytical modeling of resonance behavior.

4. Mechanical stability of the encapsulating elastomer under large deformation.

4.1 Mechanical formulation: in-plane stretch under inflation

To demonstrate that the device is not only acoustically stable but also mechanically robust, we derive the in-plane membrane stretch under inflation using the same spherical-geometry abstraction as in the Supplementary Materials (Section 2.3), and then provide complementary uniaxial tests on Ecoflex 00-30. An aperture of radius r_0 is covered by a deformable diaphragm that conforms to a spherical surface with effective radius R_s. The center of curvature partitions the deformation into two stages, and the symbol definitions follow the Note 2 to ensure consistency.

Let the undeformed membrane be a flat circular patch with area $A_0 = \pi r_0^2$. Under inflation, the deformed geometry is parameterized by (R_s, h), where $h$is the cap height measured from the diaphragm plane. As given in the Note 2 (Section 2.3), the cap height is

$$h_1 = -R_1 - \sqrt{R_{s1}^2 - r_0^2} \tag{S8}$$

$$h_2 = R_{s2} - \sqrt{R_{s2}^2 - r_0^2} \tag{S10}$$

and the inflation volume ΔV satisfies

$$\Delta V = \frac{1}{6}\pi h_1(3r_0^2 + h_1^2) \tag{S7}$$

$$\Delta V = \frac{4}{3}\pi R_{s2}^3 - \frac{1}{6}\pi h_2(3r_0^2 + h_2^2) \tag{S9}$$

Consistent with the Note 2 convention, the stretched membrane patch differs by stage. In Stage 1, the effective stretched area equals the small spherical cap:

$$S_{mem}^1 = -2\pi R_{s1} h_1 \tag{S16}$$

In Stage 2, the stretched patch corresponds to the opposite side of the sphere bounded by the same circular aperture, i.e., the full sphere minus the small cap:

$$S_{\mathrm{mem}}^2 = 4\pi R_{s2}^2 - 2\pi R_{s2} h_2 \tag{S17}$$

The equi-biaxial in-plane stretch is then defined by the area ratio

$$\lambda = \sqrt{\frac{S_{\mathrm{mem}}}{A_0}} \tag{S18}$$

Applying this pipeline, the maximum inflation $\Delta V = 100$ mL lies in Stage 2 and gives $\lambda \approx 3.47$, that is an in-plane equi-biaxial engineering strain $\lambda - 1 \approx 247\%$(area stretch $\lambda^2 \approx 12.0$). The

typical operating volume $\Delta V = 30$ mL also lies in Stage 2 and gives $\lambda \approx 2.235$, that is $\lambda - 1 \approx$ 123.5% (area stretch $\lambda^2 \approx 5.00$). These geometric results are used below to define usage-aligned cyclic tests.

4.2 Design-lifetime estimation and usage scenarios for emission and sensing

To estimate the device's mechanical lifetime, the total number of inflation–deflation cycles can be expressed as

$$N_{\text{life}} = u \times d \times L \tag{S19}$$

where u is the average cycles per day, d is the operational days per year, and L is the design lifetime in years. We distinguish two usage scenarios tied to our applications.

(1) Typical operation around 30 mL (emission: intermittent selective amplification; sensing: brief auscultatory acquisition).

A practical profile of $u = 10$–30 cycles per day, $d = 250$–300 days per year, and $L \approx 2$–3 years corresponds to $N_{\text{life}} \approx 5 \times 10^3$–$2.7 \times 10^4$. The geometric analysis gives $\lambda \approx 2.235$ at $\Delta V = 30$ mL, so we select a conservative uniaxial cyclic strain of 150% to represent this usage range in extended cycling.

(2) Infrequent extremes at 100 mL (upper-bound operation).

A conservative profile of $u = 1$–3 cycles per day, $d = 250$–300 days per year, and $L =$ 2–3 years gives $N_{\text{life}} \approx 5 \times 10^2$–$2.7 \times 10^3$. The geometric analysis gives $\lambda \approx 3.47$ at $\Delta V =$ 100 mL, so we select a conservative uniaxial cyclic strain of 300% to represent the upper-bound operating condition.

4.3 Mechanical testing aligned with usage and geometry

All specimens are ASTM-D412-C dumbbells. Tests were conducted to align with the above usage scenarios and geometric stretches.

(1) 150% strain to represent the typical 30 mL range: a specimen underwent 10,000 uniaxial loading-unloading cycles at 150% strain. Fig. S24A presents the stress–strain curves over 10,000 cycles.

(2) 300% strain to bracket the upper-bound 100 mL case: a specimen completed 1,000 uniaxial cycles at 300% strain. Representative loops at cycles 1, 50, 100, 500, and 1,000 are shown in Fig. S24B.

(3) 850% strain as the instrument stroke limit: three specimens were monotonically stretched to approximately 850% strain; the curves are shown in Fig. S24C and provide a high-stretch

reference envelope. In the context of the device, the estimated operating stress at maximum inflation is 108.36 kPa, which is well below the 488.85 kPa reference level observed at extreme stretches in monotonic tests. Together, the usage-aligned cyclic tests at 150% and 300% and the high-stretch envelope at 850% support a fatigue-tolerant operating regime for practical emission and sensing.

These estimates are mapped to results already obtained in this work: the device maintains stable acoustic output in a 100-cycle inflation-deflation test (Fig. S4), the resonant frequency remains stable over 180 minutes in both emission and sensing protocols (Figs. 2G and 3E), and week-scale tracking at fixed inflation (180 minutes per day for seven consecutive days in both modes) shows no systematic drift under identical excitation and readout conditions (Fig. S3). Independent studies on Ecoflex (including Shore 00-30) report hallmarks of cyclic durability across practical strains: an initial Mullins softening followed by stabilized unloading-reloading loops, decreasing per-cycle hysteresis, and modest rate/temperature sensitivities once conditioned. Large-deformation uniaxial tests (200-500% and higher) show repeatable responses after brief preconditioning. These findings, together with our preloaded stress-strain measurements, support Ecoflex 00-30 as fatigue-tolerant for repeated operation at the conservatively chosen strain amplitudes in this work (*79*).

Supplementary Figures

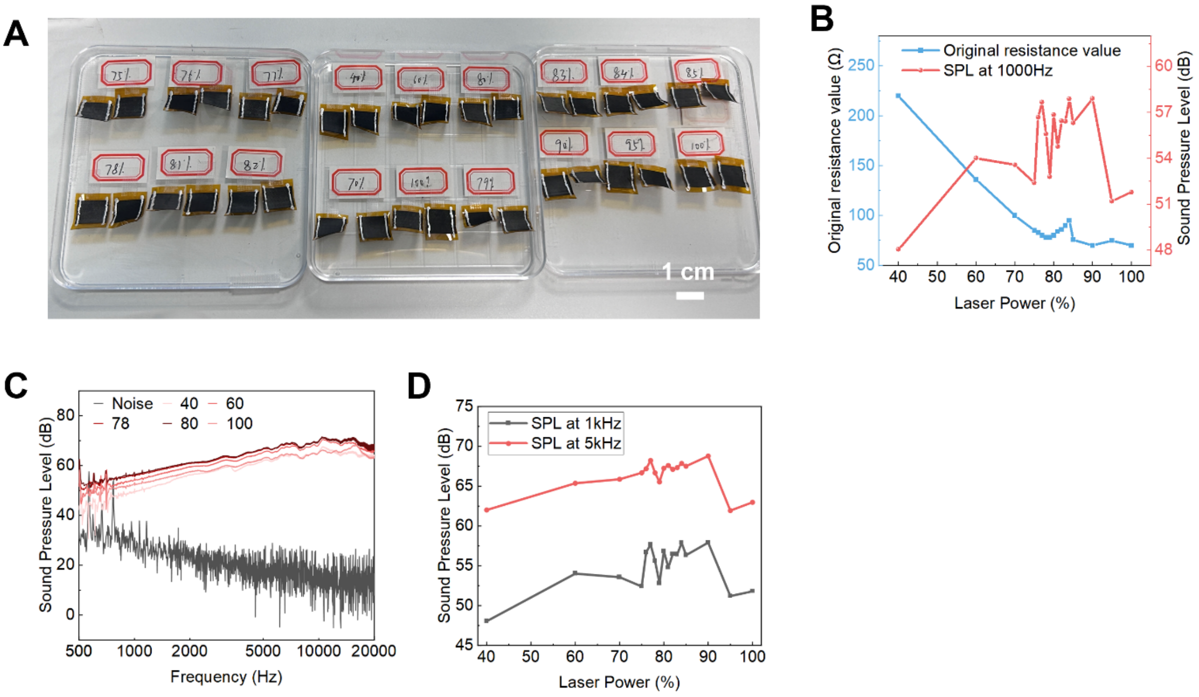

Fig. S1. Effect of laser power (1.2 W–3 W) on the performance of LIG. (**A**) Photograph of LIG with silver paste applied at the edges; the sample measures 1.2 cm×1 cm. Scale bar: 1 cm. (**B**) Relationship between the original resistance of LIG and its SPL under a 1 kHz sinusoidal input signal. Test conditions: 10 Vrms input voltage, and 5 mm distance from the microphone. At approximately laser power of 2.34 W, the LIG exhibits both lower resistance and higher SPL. (**C**) Frequency response curve of LIG, showing the highest SPL at laser power of 2.34 W. (**D**) SPL of LIG at 1 kHz and 5 kHz, with peak values also observed laser power of 2.34 W.

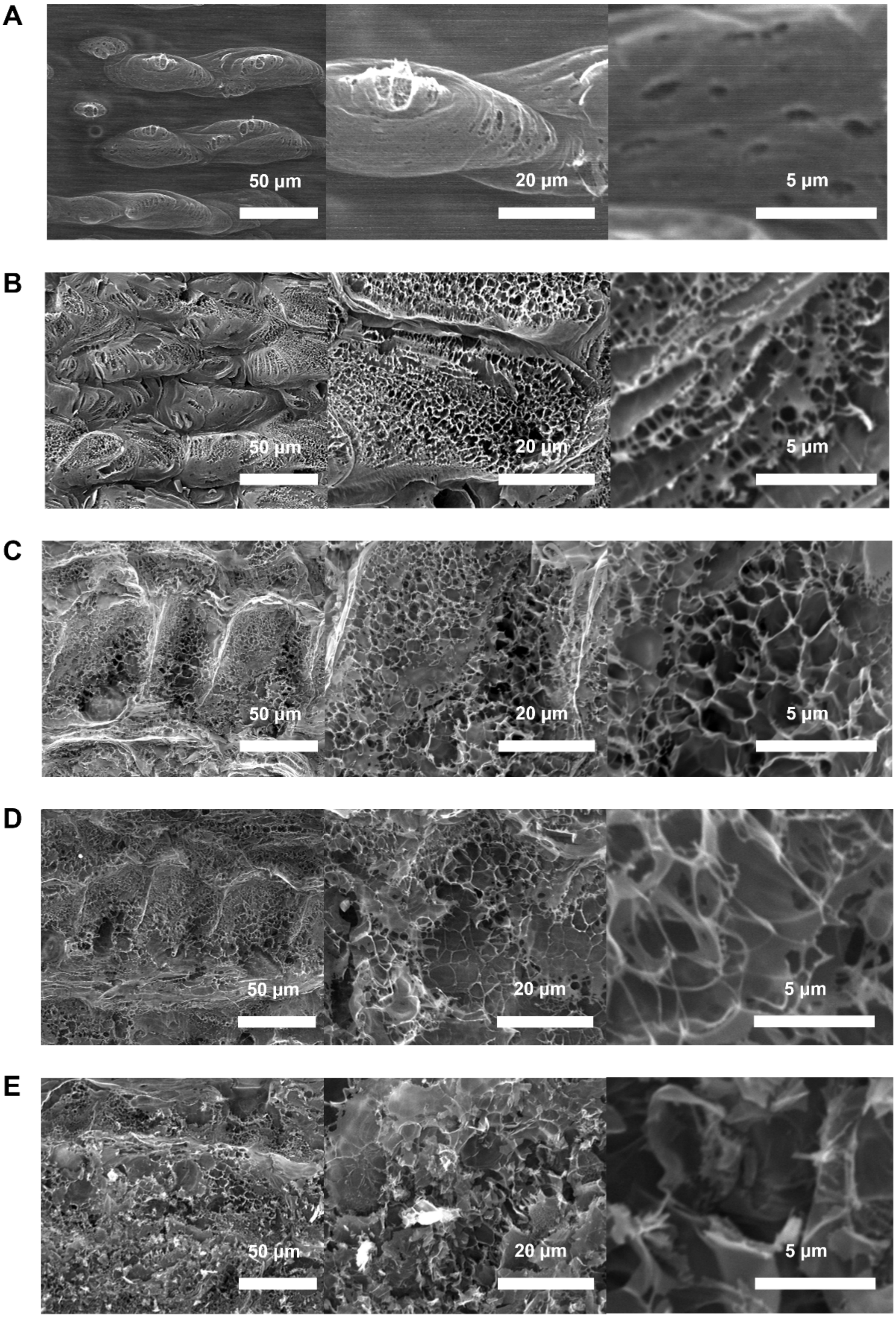

Fig. S2. SEM images of LIG fabricated under varying laser powers (0.6 W-3 W). (**A** and **B**) Under 0.6 W (**A**) and 1.2 W (**B**) laser power, the energy is insufficient for complete graphene reduction. Scale bars: 50 μm/20 μm/5 μm, consistent across all images. (**C**) Under laser power (2.34 W), graphene is well reduced. Low-magnification SEM shows periodic "gap" patterns, and high-magnification SEM reveals a clear porous morphology. This condition is thus selected for all subsequent experiments. (**D** and **E**) At 2.7 W (**D**) and 3 W (**E**), excessive energy damages previously reduced graphene, with noticeable debris visible in the SEM images.

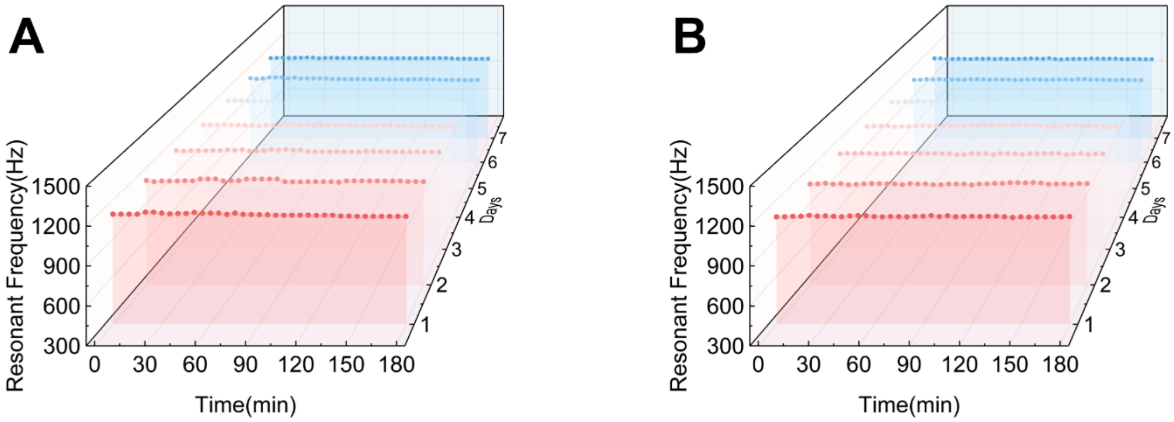

Fig. S3. Week-scale tracking of resonant frequency at a fixed inflation volume. (**A**) Emission mode. Resonant frequency vs time over 180 min per day for 7 consecutive days (Day 1–Day 7), measured under the same excitation and readout conditions as in the main-text emission tests. Each point is a single measurement; the third axis marks the day index. (**B**) Sensing mode. Resonant-frequency tracking with the same 15 mL inflation and the same protocol as in the main-text sensing tests (180 min per day, 7 days).

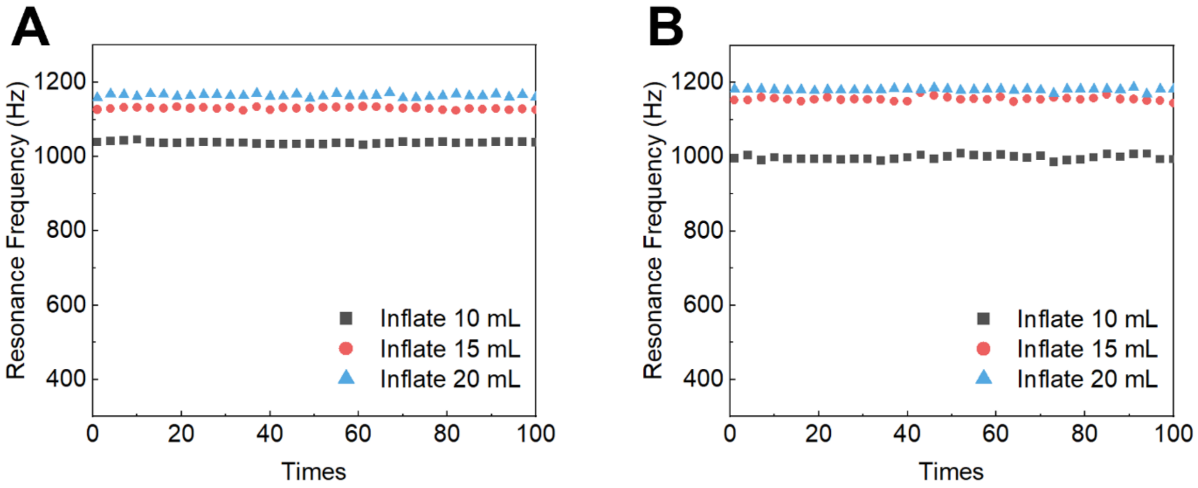

Fig. S4. Reusability test of the RAGSD. (**A**) Resonance frequency stability in the sound emission mode during 100 inflation–deflation cycles at 10 mL, 15 mL, and 20 mL inflation volumes. (**B**) Resonance frequency stability in the sound sensing mode during 100 inflation–deflation cycles at 10 mL, 15 mL, and 20 mL inflation volumes.

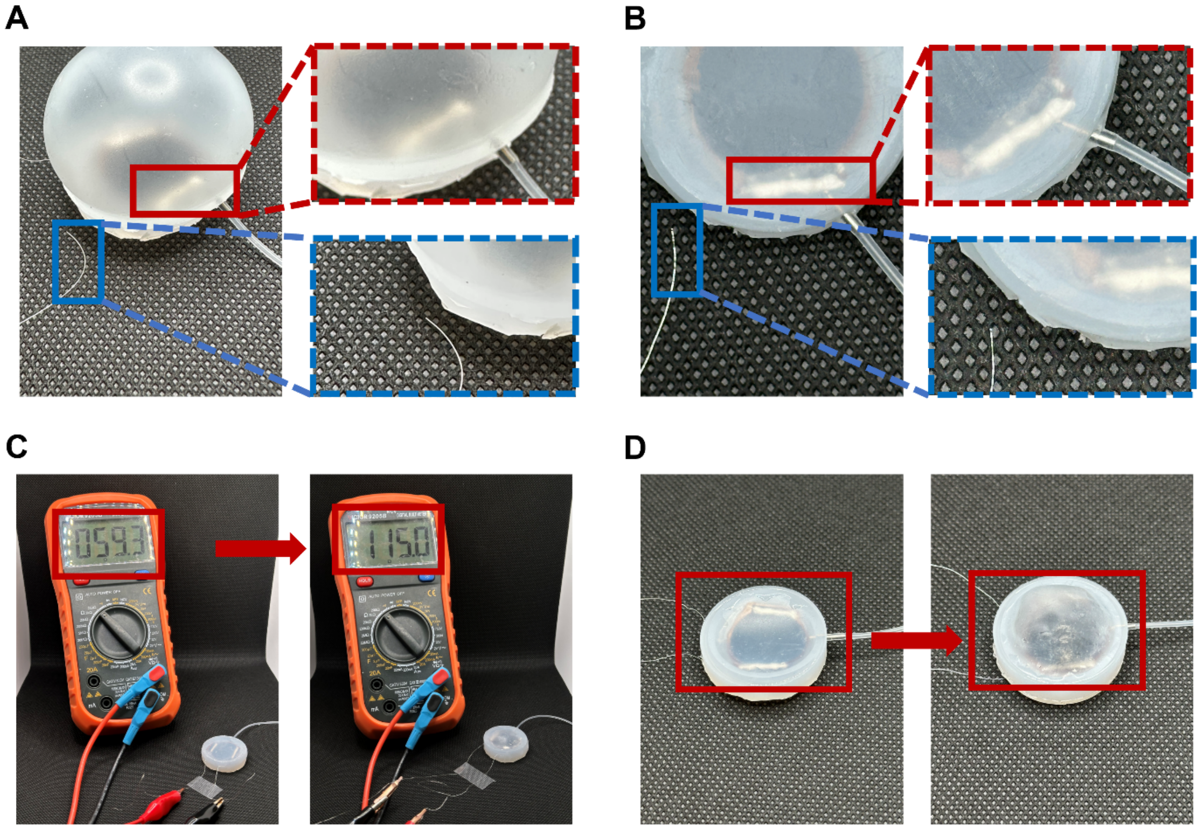

Fig. S5. Maximum inflation volume test for the RAGSD. (**A**) The RAGSD after multiple inflations exceeding 150 mL, showing silver paste detachment and silver wire breakage due to internal expansion. The close-up images highlight the affected areas. (**B**) The RAGSD after multiple inflations above 150 mL followed by deflation, showing silver paste detachment and silver wire breakage in the deflated state. Detailed images of the affected areas are shown. (**C**) Resistance measurements showing abrupt changes after repeated inflations exceeding 150 mL, caused by the detachment of silver paste and breakage of silver wires. (**D**) The RAGSD after 200 mL inflation, showing irreversible deformation with excessive tensile stress on the thin top membrane, resulting in permanent damage that cannot return to its original state.

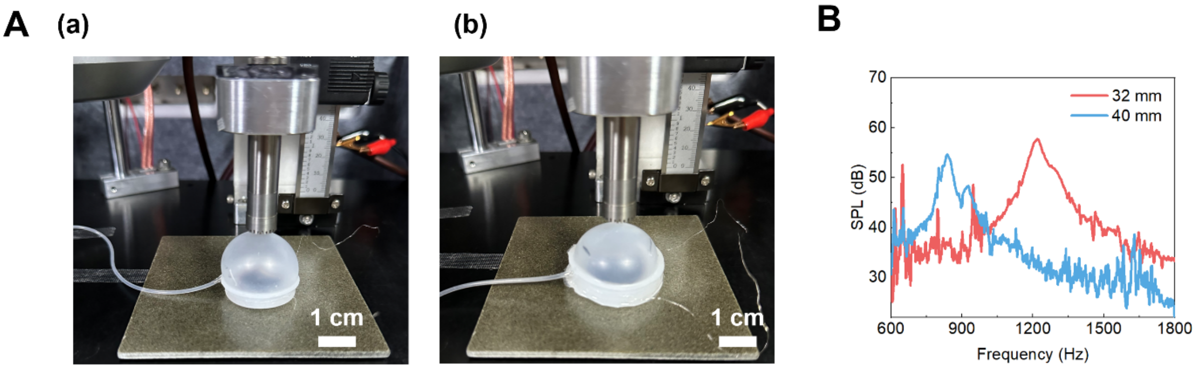

Fig. S6. Acoustic emission performance of devices with lower resonant frequencies. (**A**) Photograph of the sound emission test setup. Scale bar: 1 cm. (**a**) RAGSD with 32 mm diameter; (**b**) RAGSD with 40 mm diameter. (**B**) Frequency response curves of 32 mm and 40 mm diameter RAGSD devices under 15 mL inflation. The distance between the LIG membrane and the microphone was 2.82 cm, and the input voltage was 14 Vrms.

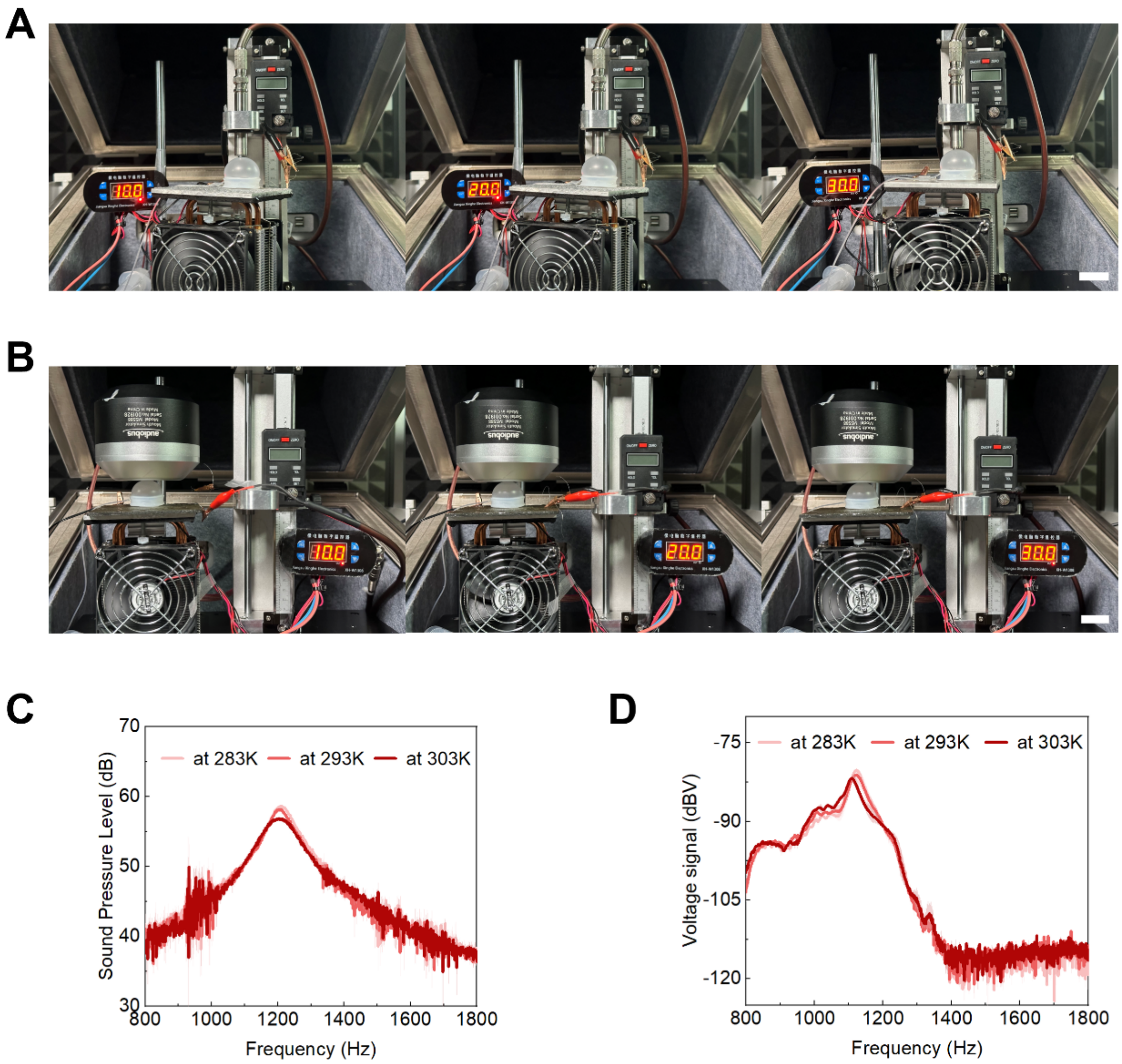

Fig. S7. Temperature dependence of the acoustic emission and sensing of the RAGSD. (**A**) Photograph of the emission setup at 283 K, 293 K, and 303 K. The RAGSD was inflated to 15 mL and driven with a 0.8-1.8 kHz swept sine at an electrical input power of 3.27 W. A free-field microphone was placed 3 cm in front of the device. Scale bar: 3 cm. (**B**) Photograph of the sensing setup at the same temperatures. An external loudspeaker provided a 0.8-1.8 kHz swept sine at 2 Vrms; the distance from the speaker to the bottom surface of the RAGSD was 3 cm. Scale bar: 3 cm. (**C**) Emission spectra measured under the conditions described in (A). (**D**) Sensing spectra measured under the conditions described in (B).

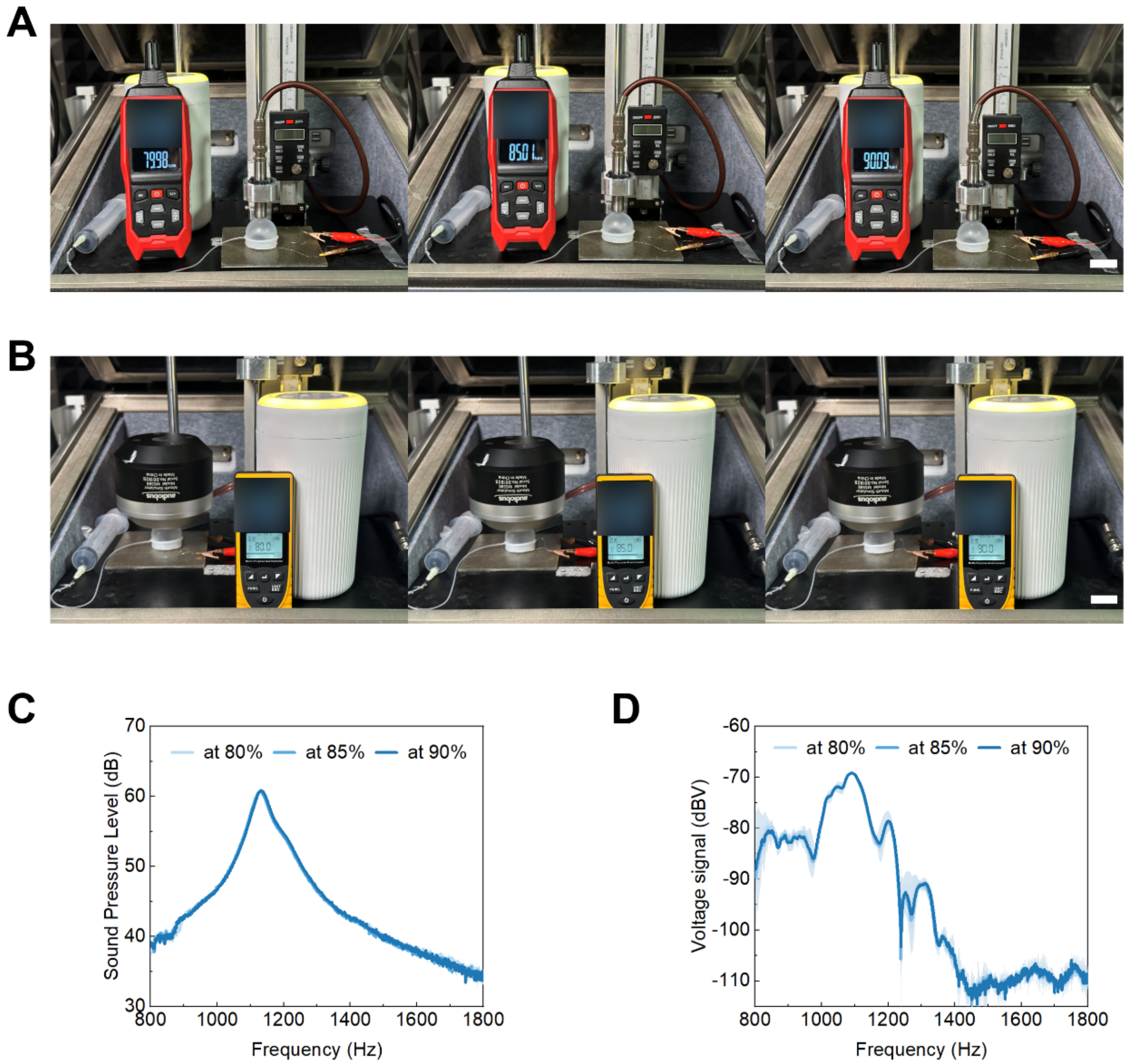

Fig. S8. Humidity dependence of the acoustic emission and sensing of the RAGSD. (A) Photograph of the emission setup at 80% Relative Humidity (RH), 85% RH, and 90% RH. The RAGSD was inflated to 15 mL and driven with a 0.8-1.8 kHz swept sine at an electrical input power of 3.27 W. A free-field microphone was placed 3 cm in front of the device. Scale bar: 3 cm. **(B)** Photograph of the sensing setup at the same humidities. An external loudspeaker provided a 0.8-1.8 kHz swept sine at 2 Vrms; the distance from the speaker to the bottom surface of the RAGSD was 3 cm. Scale bar: 3 cm. **(C)** Emission spectra at 80% RH, 85% RH, and 90% RH under the conditions in (A). **(D)** Sensing spectra at 80% RH, 85% RH, and 90% RH under the conditions in (B).

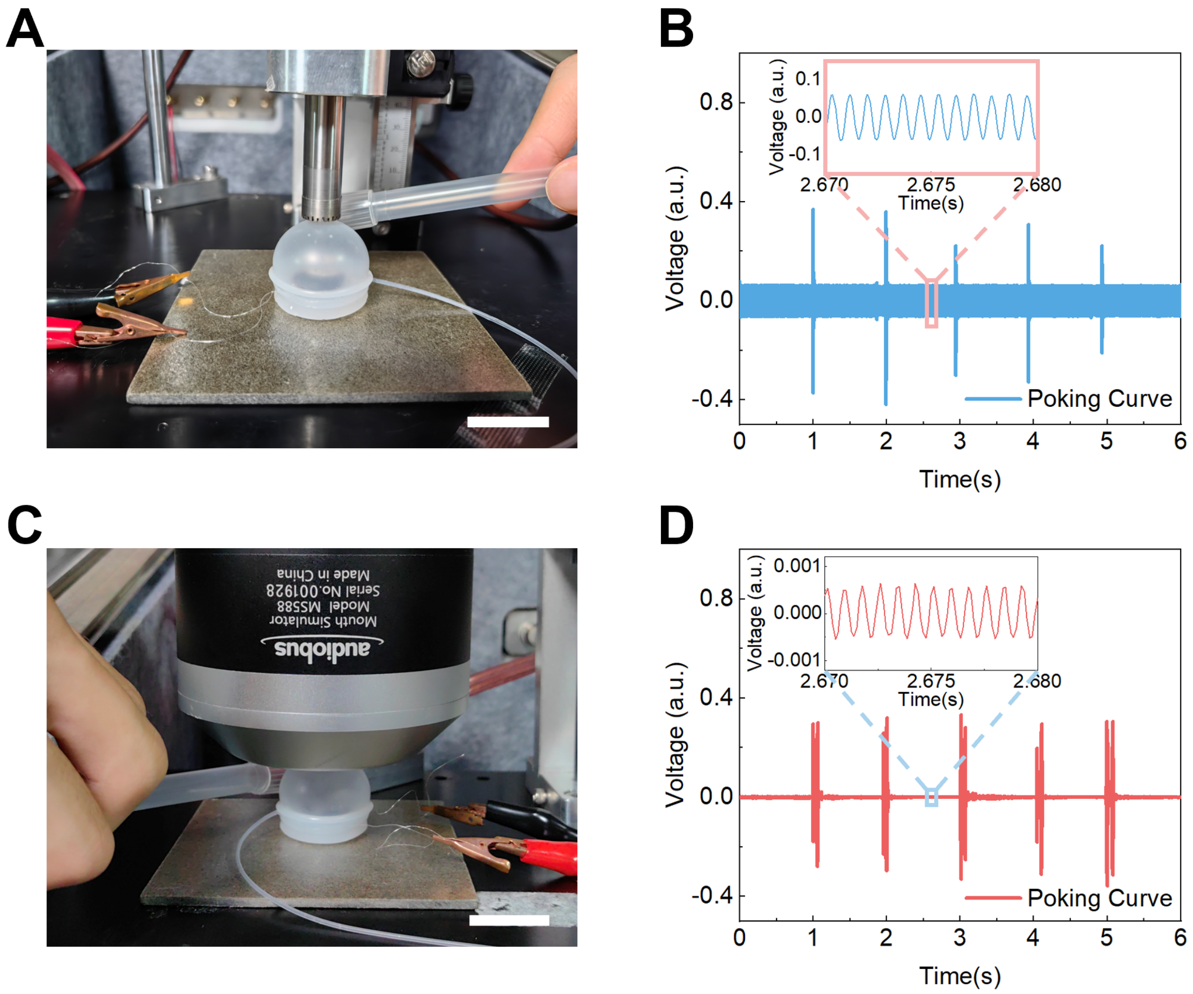

Fig. S9. Poking tests on the RAGSD under emission and sensing modes. (A) Photograph of the emission setup with manual poking. The RAGSD was inflated to 15 mL and driven by a 1.2 kHz sinusoid (electrical input power 3.27 W). **(B)** Time-domain emission signal over 6 s with five pokes applied at 1 s intervals under the conditions in (A); inset shows a zoomed window (2.670-2.680 s). **(C)** Photograph of the sensing setup with manual poking. An external loudspeaker generated a 1.2 kHz sinusoid at 2 Vrms; the distance from the speaker to the bottom surface of the RAGSD was 3 cm. **(D)** Time-domain sensing signal over 6 s with five pokes at 1 s intervals under the conditions in (C); inset shows a zoomed window (2.670-2.680 s).

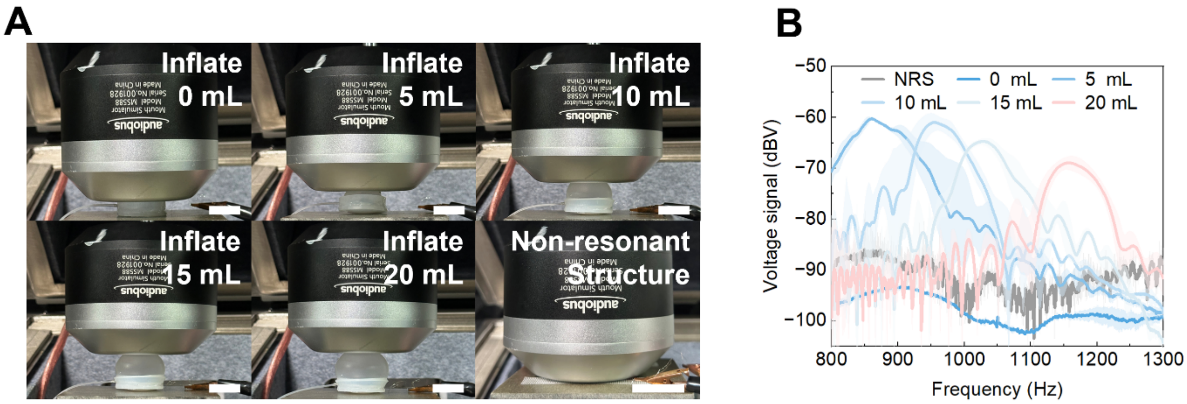

Fig. S10. Tuning of RAGSD under direct-contact conditions. (**A**) Photographs of the RAGSD under different inflation volumes (0, 5, 10, 15, and 20 mL) and Non-resonant structure (NRS) during direct contact with a surface. Scale bar: 2 cm. (**B**) Voltage signal response versus frequency for different inflation volumes and NRS, with a standard speaker input voltage of 2 Vrms. The voltage signals recorded by the device show a shift in resonance frequency with increasing inflation volume.

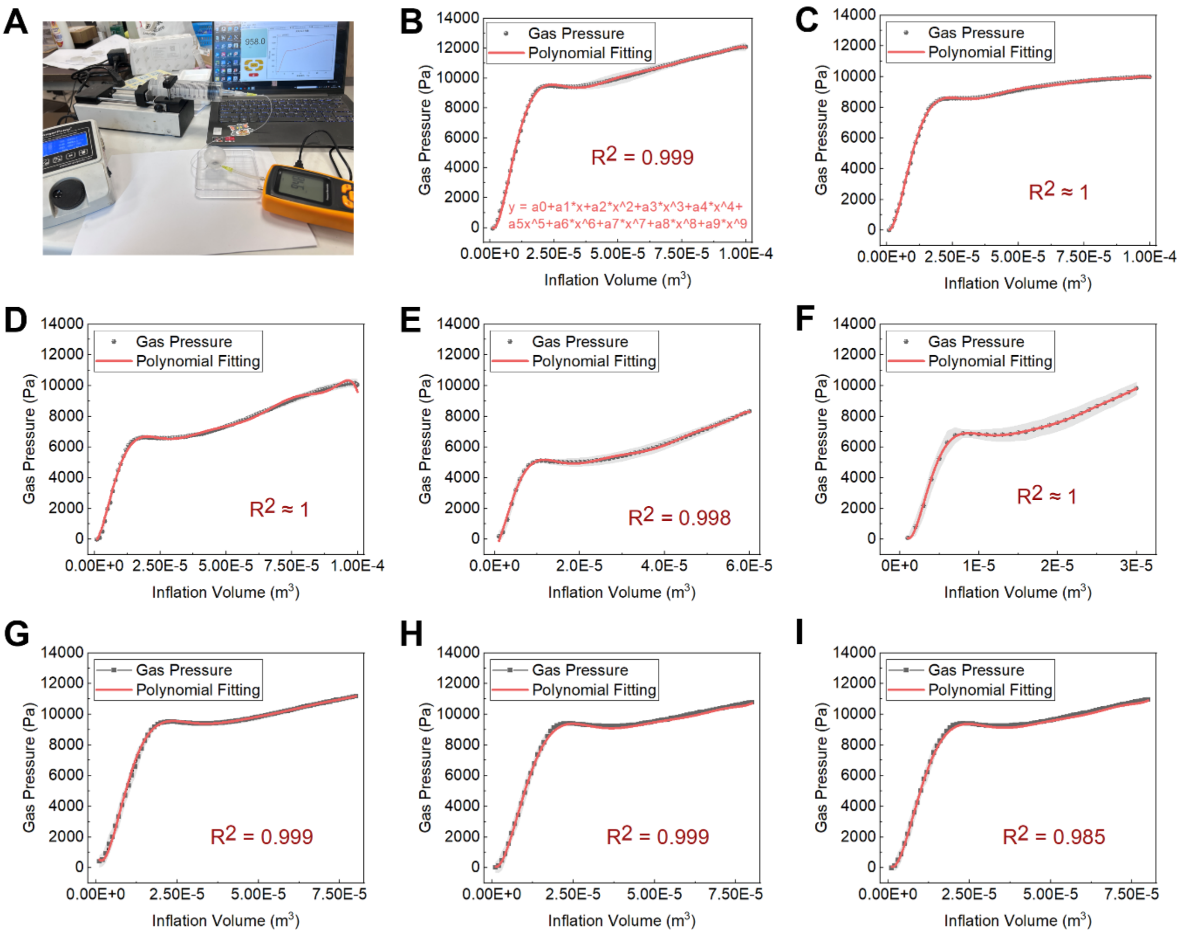

Fig. S11. Internal pressure measurement of the resonant cavity. (**A**) Photograph of the experimental setup. (**B**) Pressure-volume relationship for air inflation, showing experimental data and polynomial fitting (Poly, used throughout), with a coefficient of determination of R^2=0.999. (**C** and **D**) Pressure-volume curves for membranes with different thicknesses: 1 mm (**C**) and 0.2 mm (**D**). (**E** and **F**) Pressure-volume curves for membranes with different surface areas: diameter 26 mm (**E**) and 20 mm (**F**). (**G** to **I**) Pressure-volume curves using different inflation gases: He (**G**), N_2 (**H**), and CO_2 (**I**).

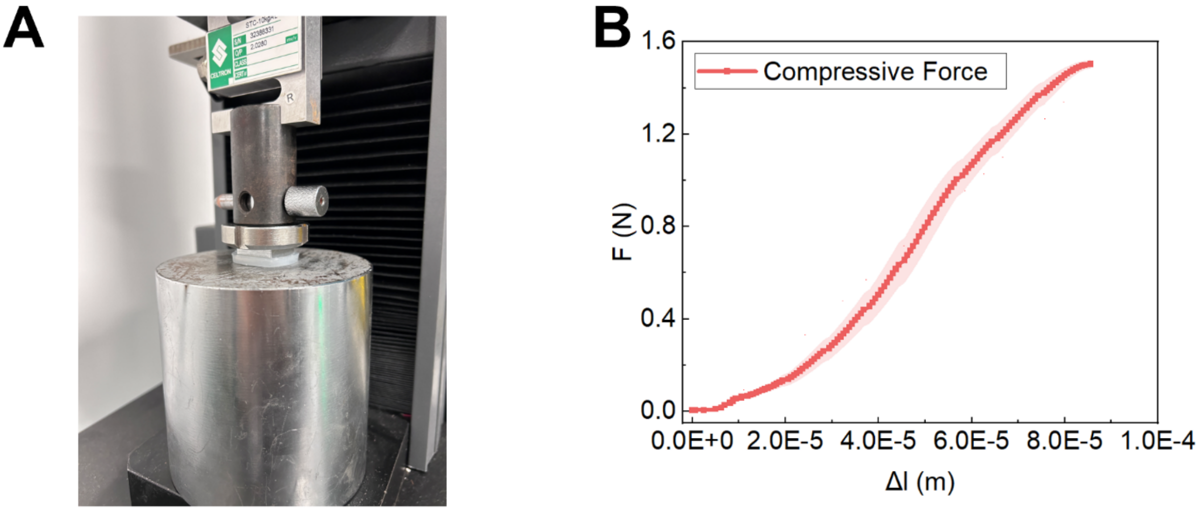

Fig. S12. Uniaxial compression test using a universal testing machine. (**A**) Experimental setup photograph. (**B**) The elastic coefficient k_m is obtained from the slope of the pressure-strain curve, with a fitted value of k_m=18181.82.

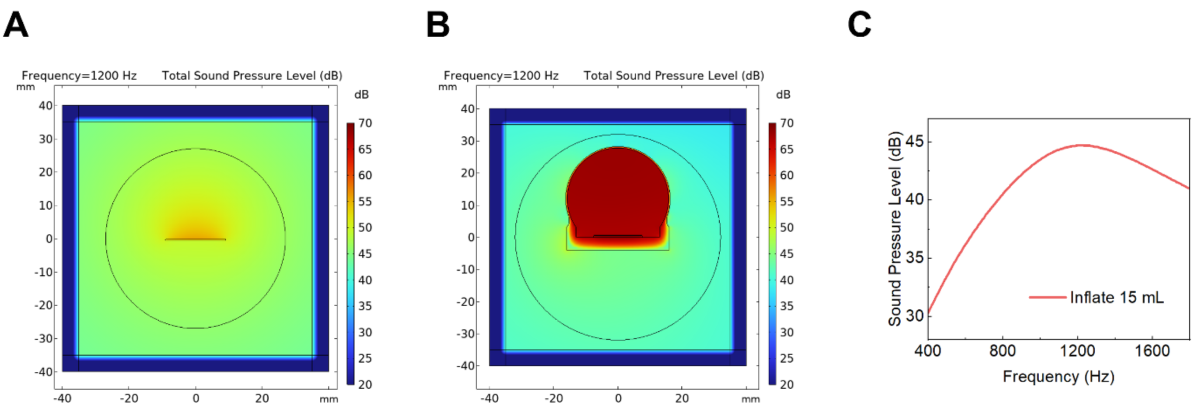

Fig. S13. Simulation results of RAGSD sound emission. (**A**) Simulated sound pressure distribution at 1.2 kHz for a flat LIG membrane without a resonant cavity. The input electrical power applied to the graphene is 1.67 W (same below). (**B**) Simulated sound pressure distribution at 1.2 kHz for the RAGSD inflated to 15 mL. (**C**) Simulated frequency response of the RAGSD under 15 mL inflation.

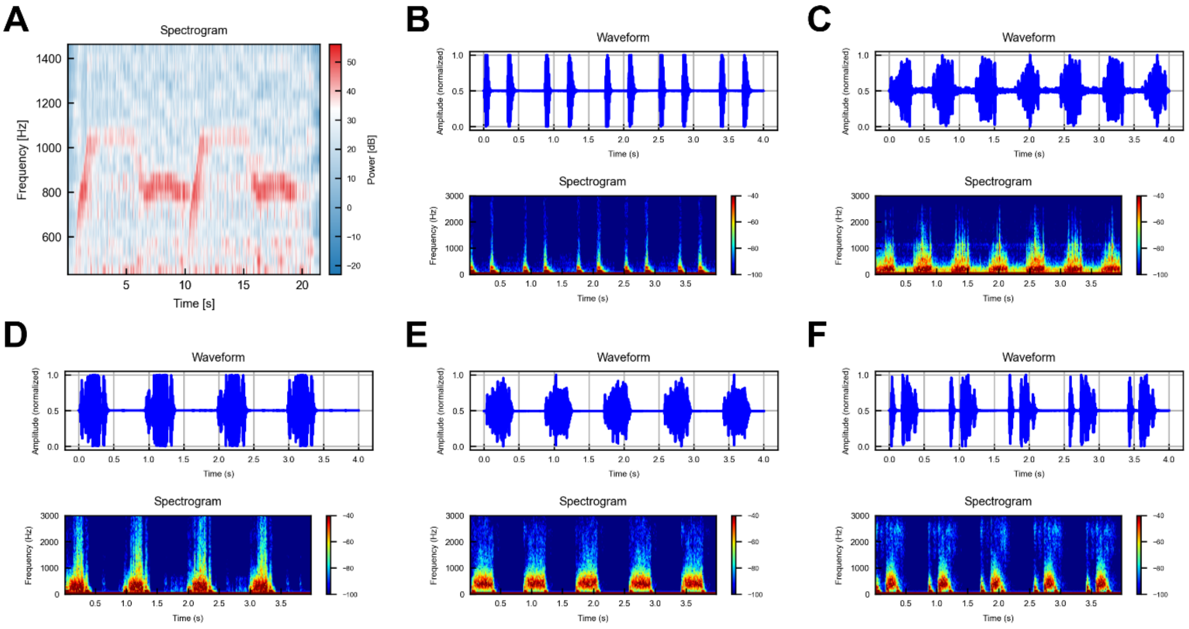

Fig. S14. Spectrogram analysis of audio signals used in this study. (**A**) Spectrogram of the original audio clip from *Opera No. 2*. (**B** to **F**) Normalized time-domain waveforms and frequency spectra of representative heart sounds: normal heart sound (N) (**B**), ventricular septal defect (VSD) (**C**), aortic stenosis (AS) (**D**), mitral regurgitation (MR) (**E**), mitral valve prolapse (MVP) (**F**).

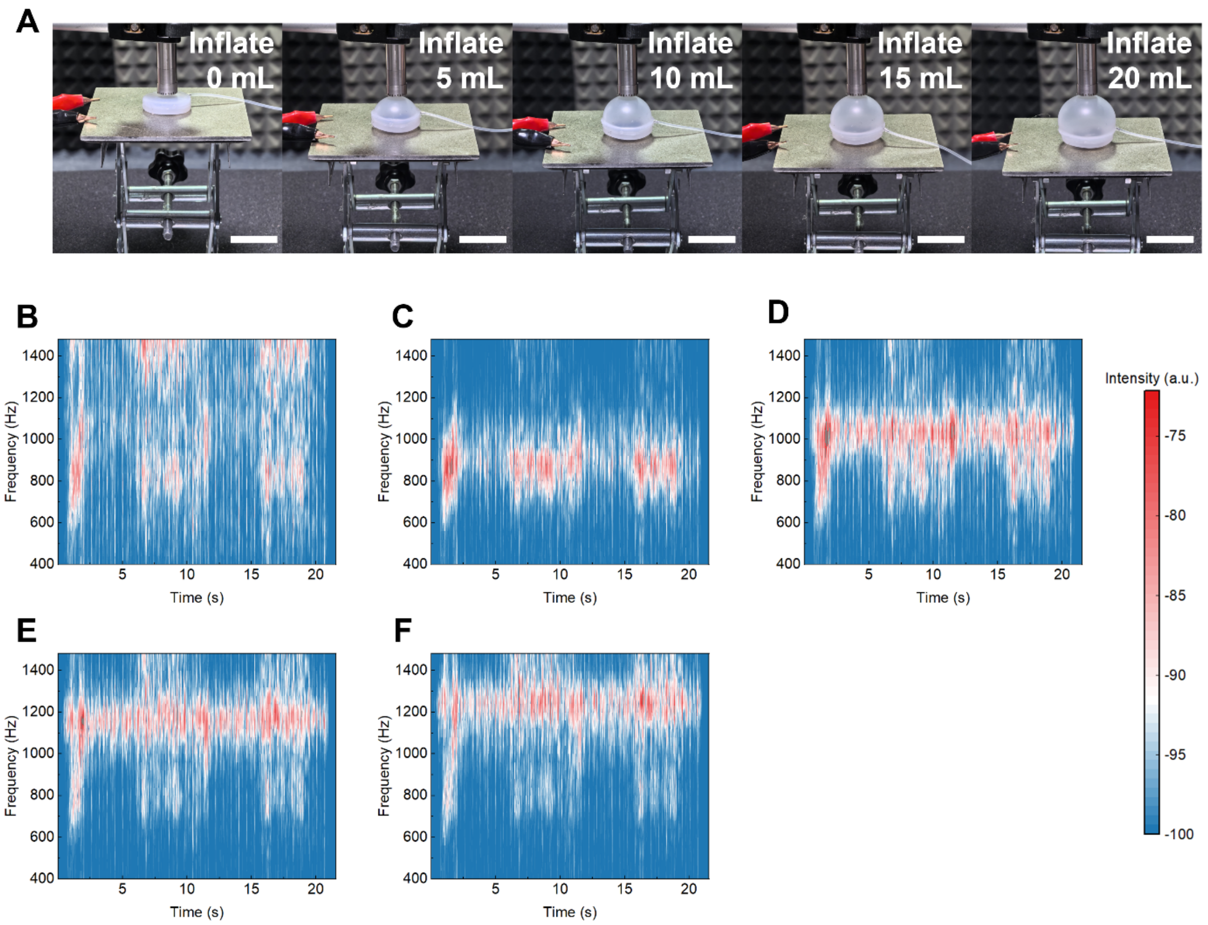

Fig. S15. Control study on emission with fixed membrane–microphone spacing. (**A**) Photographs of the RAGSD under different inflation volumes of 0, 5, 10, 15, and 20 mL, showing the experimental setup with a constant membrane–microphone distance of 1 mm. (**B** to **F**) Spectrograms of RAGSD acoustic emission recorded driven by an AC-modulated audio waveform (chorus segment of *Opera No. 2*) at inflation volumes of 0 mL (**B**), 5 mL (**C**), 10 mL (**D**), 15 mL (**E**), and 20 mL (**F**) under a constant input power of 3.27 W.

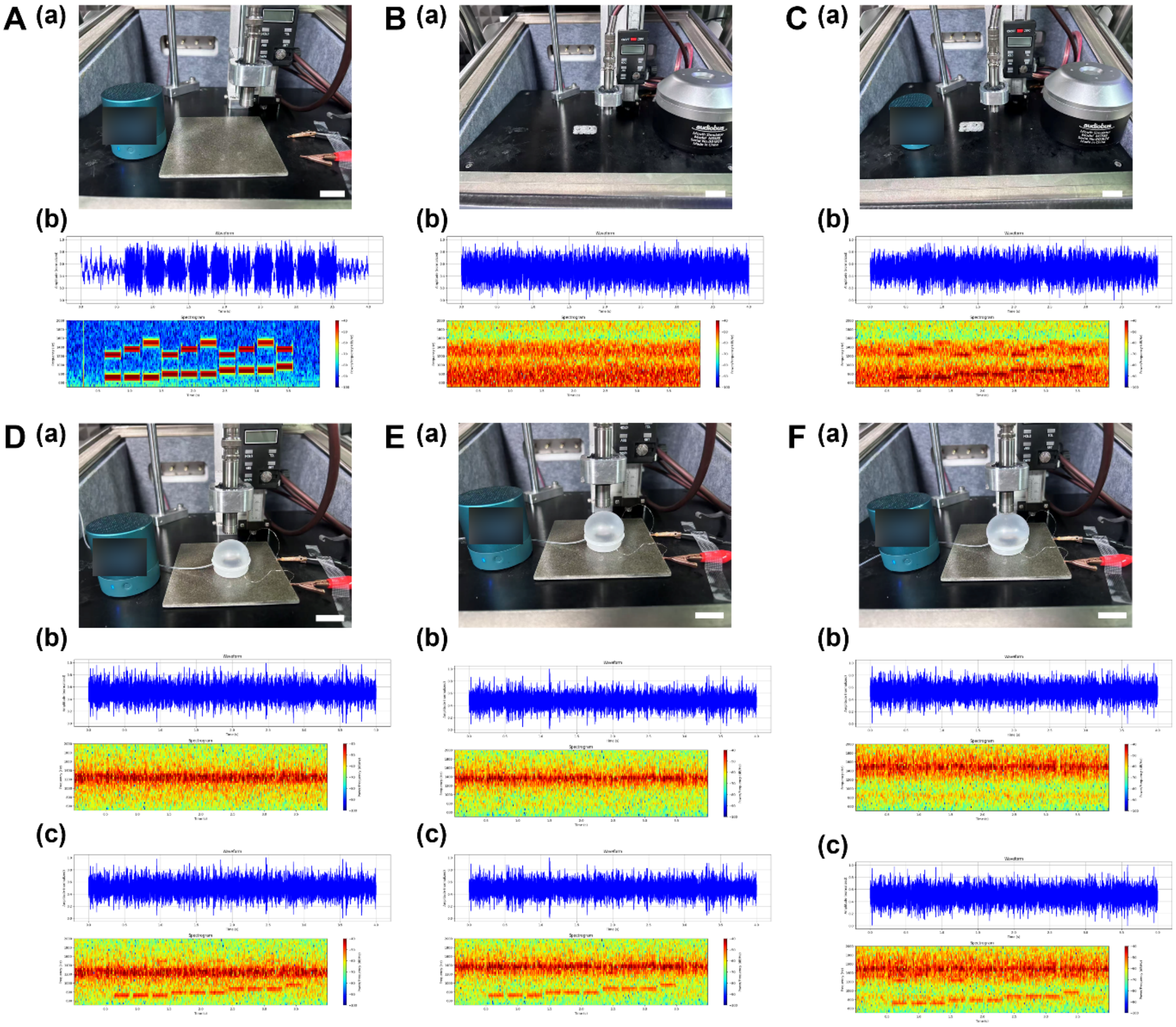

Fig. S16. Frequency-selective masking of DTMF bands using a tunable RAGSD emitter. (**A**) Target signal. The target is a DTMF example of the phone sequence "1234567890" (hereafter the same). (**a**) Photograph of the recording setup. Scale bar, 3 cm; same below. (**b**) Time-domain waveform (top) and spectrogram (bottom) of the target. (**B**) Loudspeaker control, masker only. (**a**) Photograph of the setup; the loudspeaker plays band-limited pseudo-white noise from 500 to 1500 Hz. (**b**) Time-domain waveform (top) and spectrogram (bottom) of the masker. (**C**) Loudspeaker control, target plus masker. (**a**) Photograph of the setup; simultaneous playback of the DTMF target and the 500-1500 Hz masker. (**b**) Time-domain waveform (top) and spectrogram (bottom) of the mixed signal. (**D–F**) Masking with the RAGSD under tunable emission. The device is inflated to (**D**) 16 mL, (**E**) 22 mL, and (**F**) 34 mL, placing its emission peak near 1209, 1336, and 1477 Hz, respectively. For each inflation volume: (**a**) Photograph of the setup; (**b**) masker-only recording with the RAGSD driven by 500-1500 Hz band-limited pseudo-white noise, shown as time-domain waveform (top) and spectrogram (bottom); (**c**) target plus masker recording, shown as time-domain waveform (top) and spectrogram (bottom).

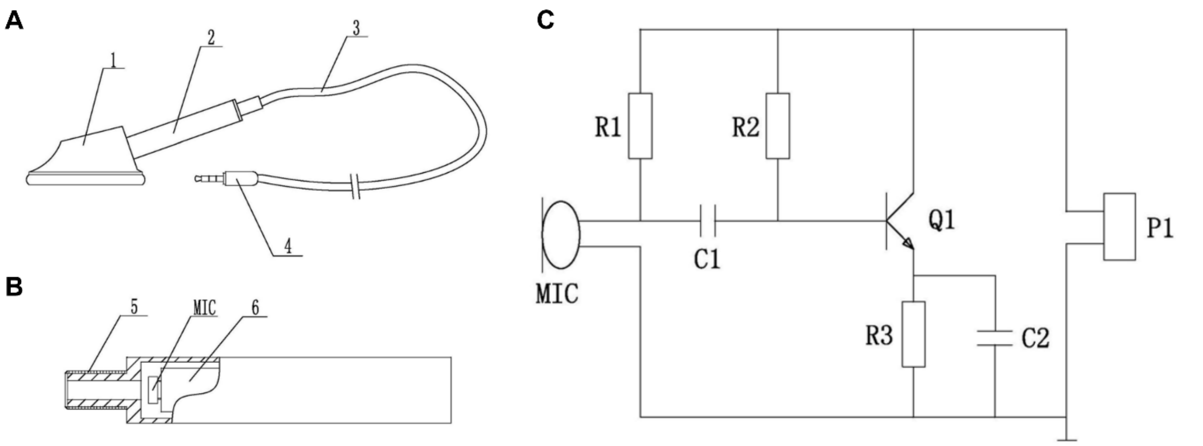

Fig. S17. Commercial electronic stethoscope and acoustic pickup module (*80*). (A) Schematic of a commercial electronic stethoscope. Labeled components: 1, auscultation probe; 2, acoustic pickup tube; 3, data cable; 4, data connector. **(B)** Structural schematic of the acoustic pickup tube. Labeled components: 5, threaded end connected to the auscultation probe; 6, signal amplification circuit board; MIC, acoustic to electric transducer. **(C)** Circuit diagram of the signal amplification module with an approximate voltage gain of four.

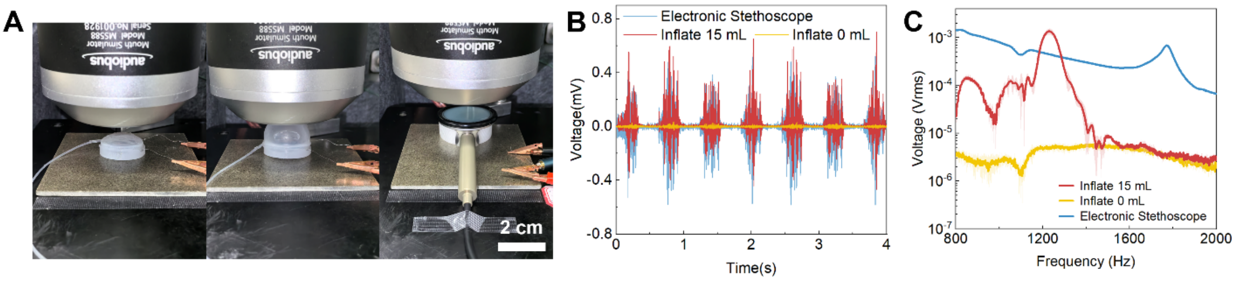

Fig. S18. Comparison of acoustic sensing performance between the RAGSD and a commercial electronic stethoscope. (**A**) Photograph of the experimental setup, showing (from left to right): uninflated RAGSD, RAGSD inflated to 15 mL, and a commercial electronic stethoscope. Scale bar: 2 cm. The loudspeaker was excited by a 2 Vrms drive voltage., and the distance from the speaker to the bottom surface of each device was 3 cm. (**B**) Time-domain signal amplitude comparison for recorded VSD heart sounds. (**C**) Frequency response comparison of the three devices.

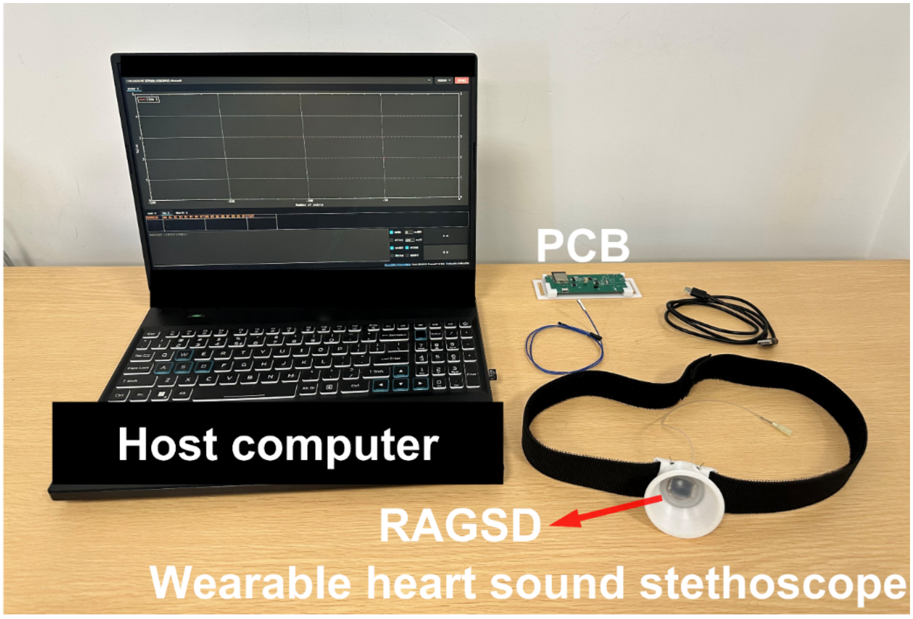

Fig. S19. Physical image of the real-time wearable intelligent heart sound monitoring system. The system includes: the core wearable heart sound stethoscope, the PCB for real-time heart sound signal acquisition with a 5 kHz high-frequency sampling rate, the main control computer displaying the pre-processed heart sound waveform data, as well as connecting cables, custom-designed enclosure, and an elastic strap for secure fixation.

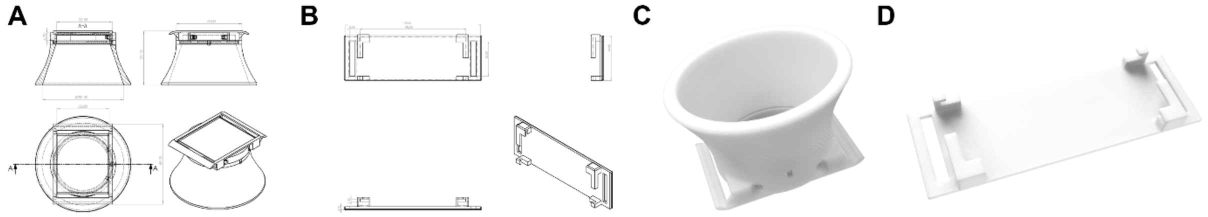

Fig. S20. Engineering design and 3D models of the RAGSD stethoscope components. (**A**) The engineering drawing of the RAGSD stethoscope cavity component. (**B**) The engineering drawing of the PCB card slot component. (**C**) The 3D model of the RAGSD stethoscope cavity. (**D**) The 3D model of the PCB card slot.

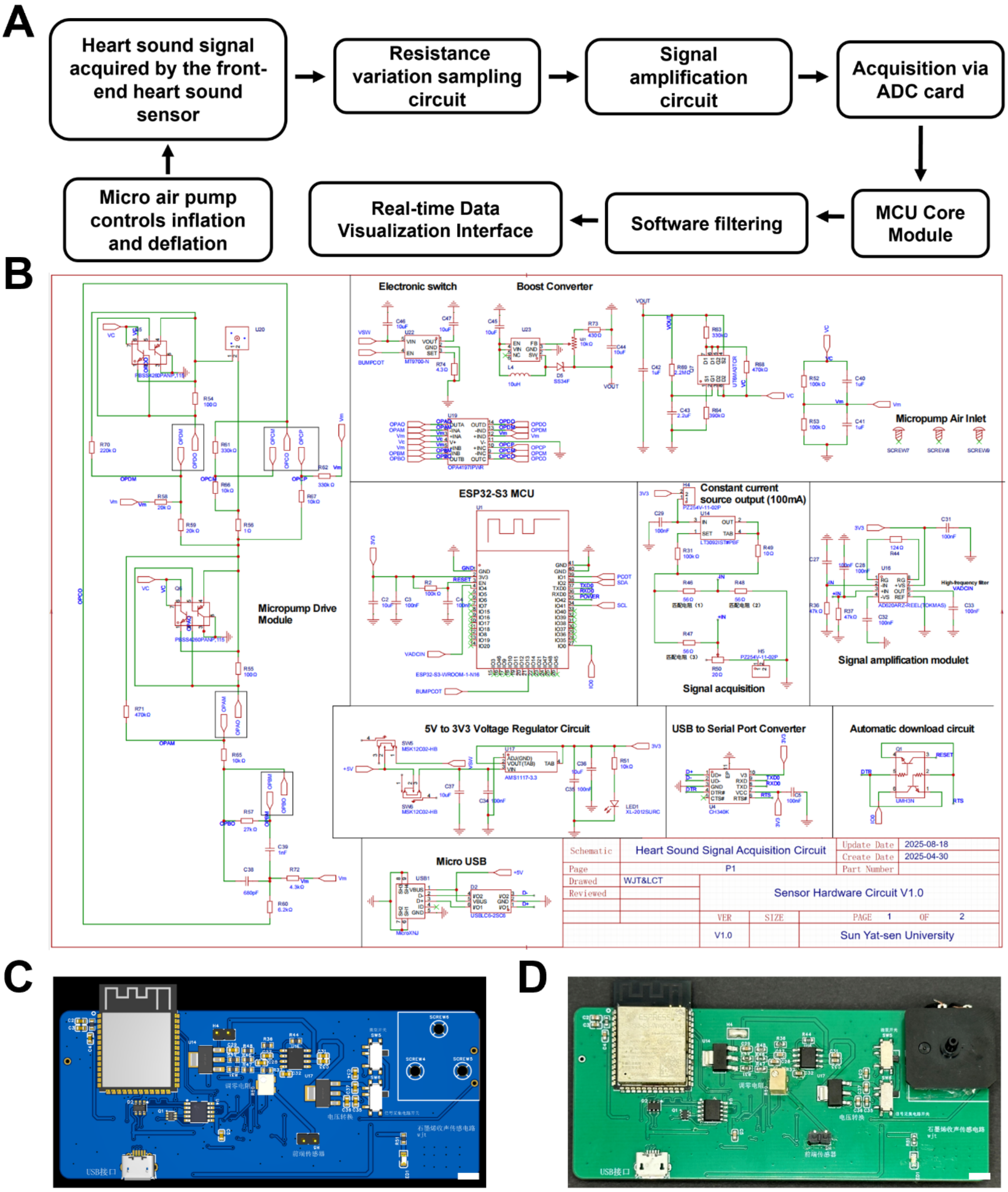

Fig. S21. Design and Results of Signal Acquisition Circuit System. (**A**) Hardware block diagram of the signal acquisition circuit. (**B**) Schematic diagram of the signal acquisition circuit. (**C**) 3D preview of the PCB enclosure of the signal acquisition circuit. Scale bar: 0.5 cm. (**D**) Physical Image of the PCB of the signal acquisition circuit. Scale bar: 0.5 cm.

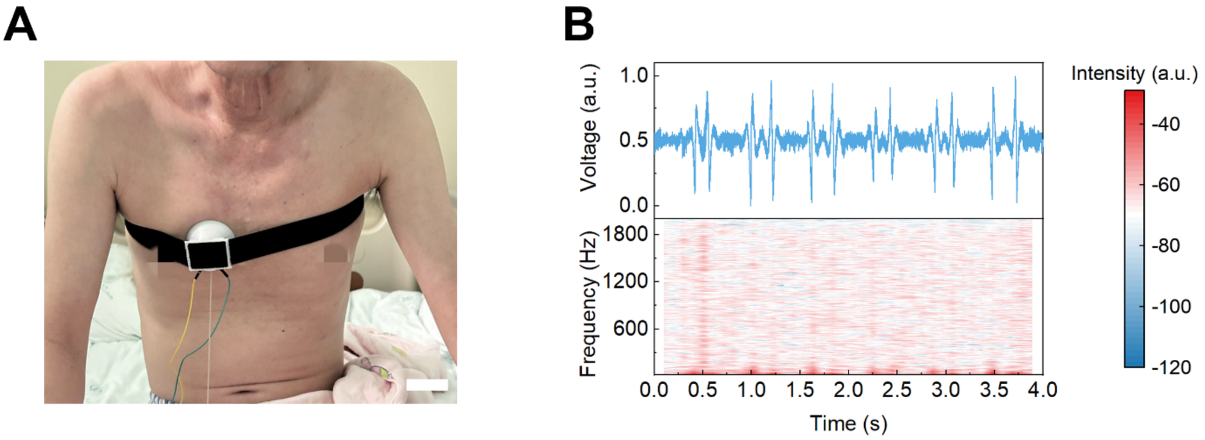

Fig. S22. Wearable RAGSD recording from a patient with aortic regurgitation. (**A**) Photograph of the RAGSD probe positioned at the aortic second intercostal space on the patient (scale bar: 5 cm). (**B**) Time-domain waveform (top) and time–frequency spectrogram (bottom) of heart sounds recorded from the mitral area of the same patient.

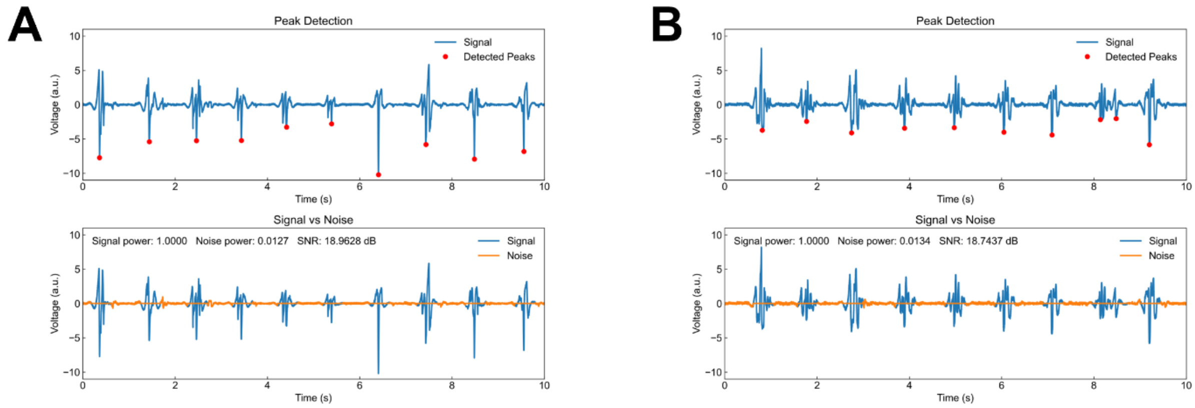

Fig. S23. On-body heart-sound signals in quiet and noisy environments. (**A**) Quiet anechoic room. The upper panel shows a 10 s waveform with detected S1/S2 peaks (red markers). The data are high-pass filtered at 5 Hz and amplitude-normalized. The lower panel shows signal windows centered on detected peaks and noise intervals taken from the remaining segments. The same processing is used for subsequent panels. (**B**) Added broadband ambient noise with identical mounting and acquisition settings. The upper and lower panels show the waveform and the corresponding signal/noise traces, respectively.

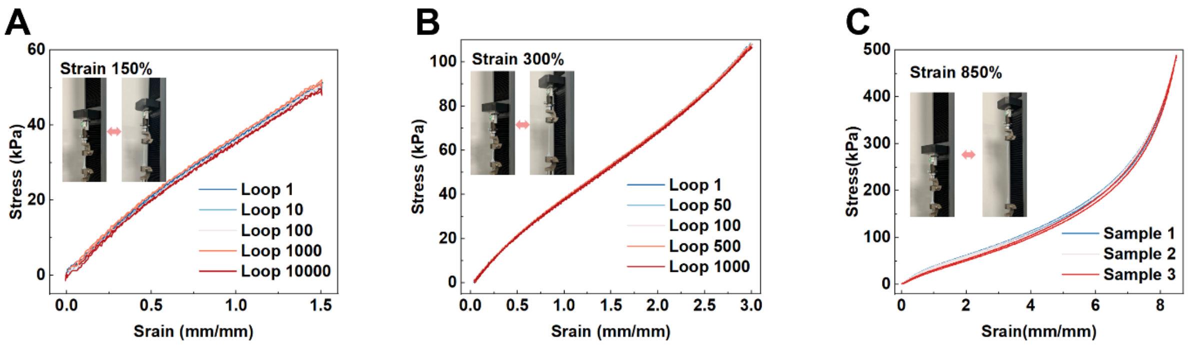

Fig. S24. Cyclic and monotonic tensile characterization of Ecoflex 00-30. (**A**) Cyclic loading-unloading to 150% strain; stress-strain loops are shown for cycles 1, 10, 100, 1000, and 10000. Insets: specimen at the initial state and at 150% stretch. Specimens follow ASTM-D412-C: test speed is 500 mm/min; Specimens were preloaded via the UTM grips to remove slack. same below. (**B**) Cyclic loading-unloading to 300% strain; stress-strain loops are shown for cycles 1, 50, 100, 500, and 1000. Insets: specimen at the initial state and at 300% stretch. (**C**) Monotonic uniaxial tension of three specimens to 850% strain; stress-strain curves for Sample 1-3. Insets: specimen at the initial state and at 850% stretch.

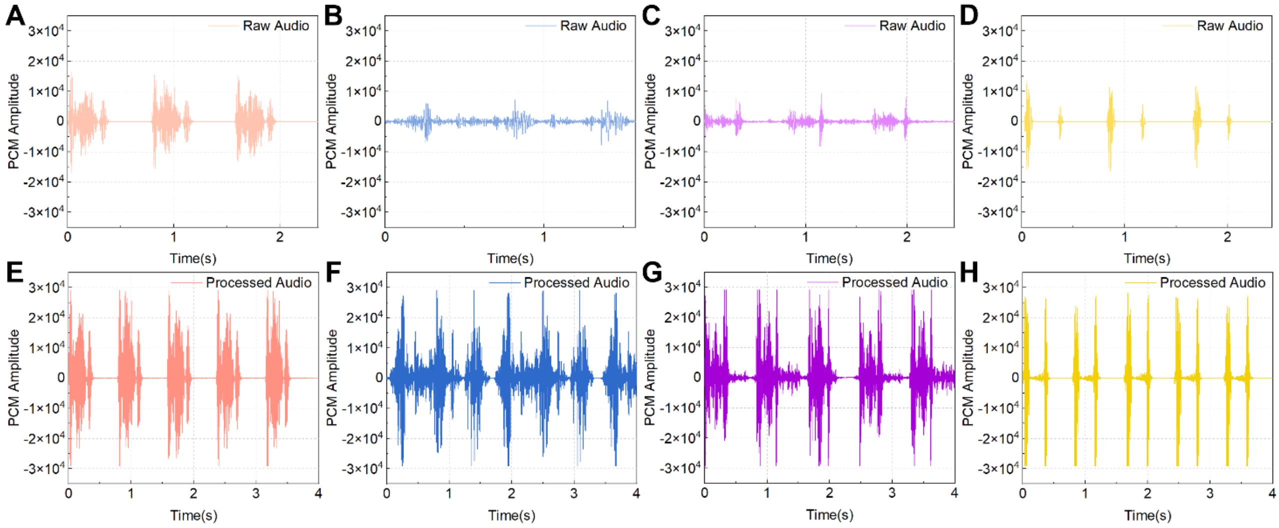

Fig. S25. Preprocessing of heart sound data from a public database. (**A** to **D**) Raw samples from four heart sound categories: AS (**A**), MR (**B**), MVP (**C**), and N (**D**), with one representative signal shown for each. (**E** to **H**) Corresponding preprocessed signals for AS (**E**), MR (**F**), MVP (**G**), and N (**H**), with one representative example shown per category.

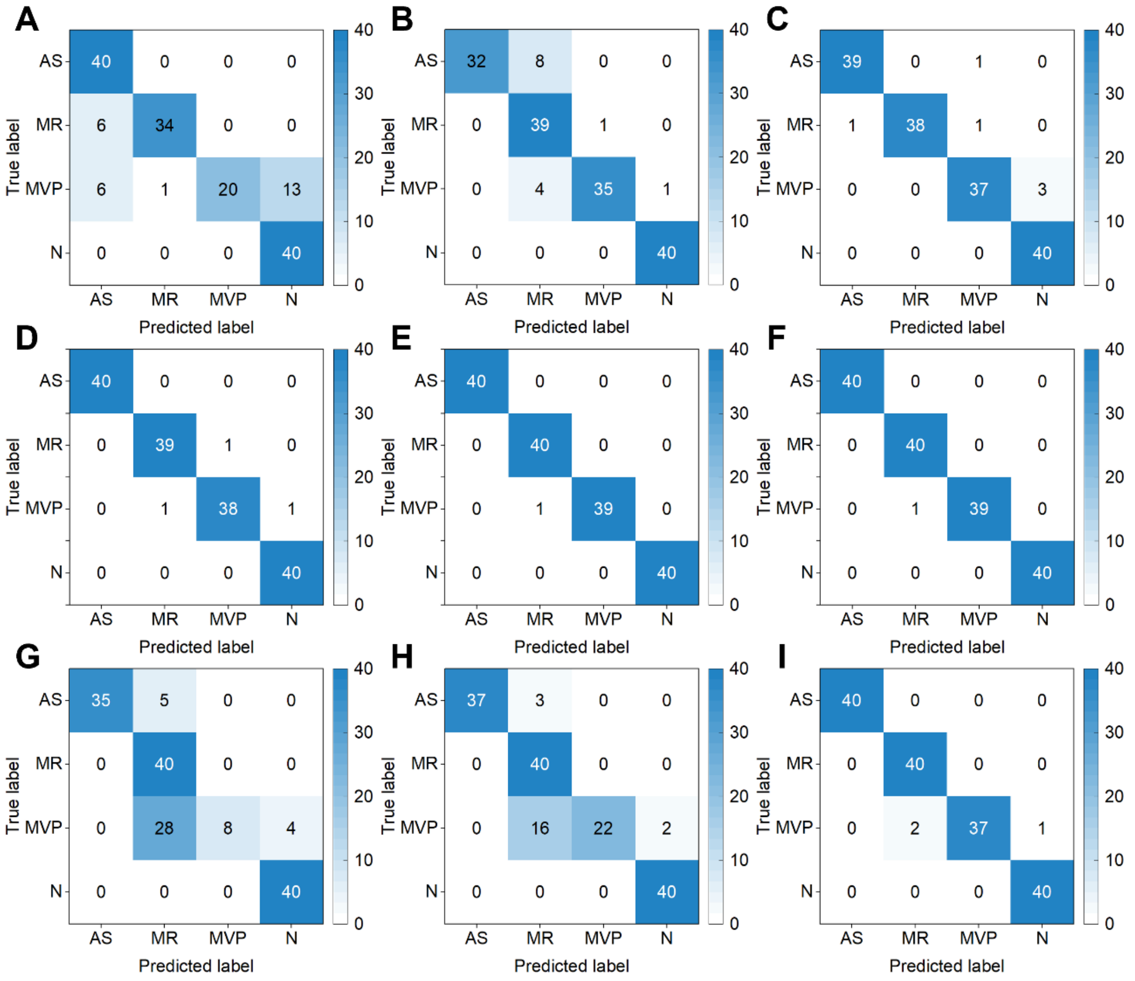

Fig. S26. Confusion matrices visualizing the training process of the AuscNet-H on different datasets and epochs. (**A** to **C**) Confusion matrices after 10 (**A**), 20 (**B**), and 30 (**C**) training epochs using the 0 mL uninflated dataset. (**D** to **F**) Confusion matrices after 10 (**D**), 20 (**E**), and 30 (**F**) training epochs using the 15 mL inflated dataset. (**G** to **I**) Confusion matrices after 10 (**G**), 20 (**H**), and 30 (**I**) training epochs using the commercial electronic stethoscope dataset.

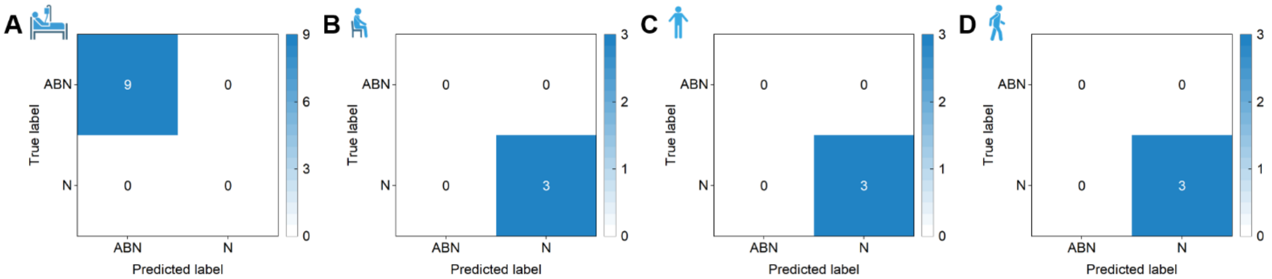

Fig. S27. Confusion matrices of the neural-network classifier on human-subject data. (**A to D**) Two-class confusion matrices with labels **ABN** and **N**; color bars indicate sample counts; identical mounting and acquisition settings; 4-s segments: aortic-regurgitation patient in supine and seated postures (**A**), healthy subject seated (**B**), standing (**C**), and slow walking (**D**).

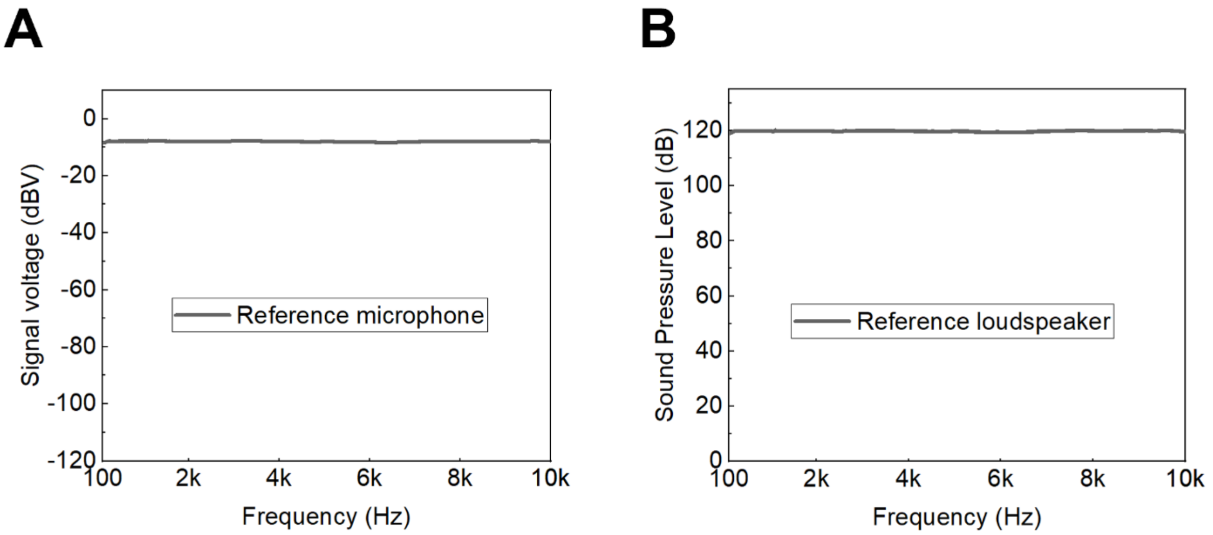

Fig. S28. Reference microphone sensitivity spectra and reference speaker frequency response. (**A**) The sensitivity spectra curve of the reference microphone measured from 100 Hz to 10 kHz, showing a flat response across the tested frequency range. (**B**) The sound pressure level curve of the reference loudspeaker, also measured from 100 Hz to 10 kHz, showing consistent sound output across the same frequency range.

Supplementary Tables

Table S1. Comparison of flexible acoustic sensors.

Name	Materials	Sensing principle	Functional highlights	Sensing performance	Intelligence	Ref.
A hydrogel-based soft mechanical sensor	Hydrogel iontronic; flexible	Ionic conduction	Broadband flat FR	Band: 0–1000 Hz; flatness: −23 dB; LOD: 1.08 Pa; SNR: 94 dB @1 kHz	Unable	(*30*)
An artificial basilar membrane (ABM)	Nanowire membrane on elastomer; flexible	Piezoresistive	BM-like selectivity	Band: 319–1951 Hz; SPL: 95–100 dB; peaks: 530/970/2250 Hz	Unable	(*31*)
Stretchable water–silicone acoustic transducer	Water/hydrogel in silicone; stretchable	Acoustic coupling layer	Through clothing/hair	N.A.	Unable	(*32*)
Basilar-membrane-inspired self-powered sensor	Flexible self-powered; flexible	Tribo/Piezo (self-powered)	Multi-tunable bands	Band: 100 Hz–4 kHz; SPL: 94 dB; LOD: 1 Pa; peaks: 650/1440 Hz	Unable	(*33*)
Frequency-selective smart skin	Triboelectric composite; flexible	Triboelectric	Band-selective array	Band: 145–9000 Hz; pressure: 2025 Pa; range: 3–70 kPa	>95% (voice)	(*34*)
Liquid acoustic throat sensor	Permanent fluidic magnet+coil; soft/liquid interface	Magnetic induction	Self-filters <30 Hz	Band: 30 Hz–10 kHz; LOD: 0.9 Pa; SNR: 69.1 dB; SPL: 90 dB	99% (voice, noisy)	(*35*)
Flexible ZnO/Al acoustic platform	ZnO thin film on Al foil; flexible	Piezoelectric (mic mode)	HMI mic-mode	Band: 20 Hz–20 kHz; levels: 76/49/17 dB; SPL: 90 dB	98% (speech)	(*36*)
Polystyrene-sphere “artificial throat”	AgNW/rGO+PS; flexible	Piezoresistive	Throat micro-vibration	LOD: 2.1 Pa; sensitivity: 21.8 kPa^{-1}; pressure: 20.6–33 Pa	>96% (5-word SR)	(*37*)
Omnidirectional tribo SAS	Porous film SAS; flexible	Triboelectric	3D stereo sensing	Band: 100 Hz–20 kHz; sensitivity: 3172.9 mV Pa^{-1};	98% (HMI task)	(*38*)

				SNR: 56.37/46.45 dB		
**RAGSD**	**All-soft elastomer; on-skin**	**Piezoresistive**	**Full-band sensing + tunable resonance**	**Band: Full-band; tunable band: 922–1763 Hz; span: 91.18%;**	**99.375% (heart-sound 4-class)**	**this work**

Table S2. Comparison of different methods for tuning resonance frequency.

Category	Name	Resonance Method	Continuous Frequency Tuning Range (Hz)	Tuning Range (%)	Reference
I. Fixed single-resonator	Graphene earphone	Fixed-shape encapsulation shell	Unable	Unable	(*20*)
	Panel–cavity thermoacoustic projectors	Cavity-diaphragm resonance	Unable	Unable	(*39*)
II. Discrete selectable resonators	Carbon nanotube (CNT) thermoacoustic projectors	Compartment filled with a gas such as Xe, Ar, or air	Unable	Unable	(*40*)
	CNT loudspeaker	Multiple discrete resonators	Unable	Unable	(*41*)
	Graphene conch-like cavity	Switching spiral cavity geometries	Unable	Unable	(*42*)
III. Continuously tunable structures	Parallel-plate actuator	Electrostatic spring effect	~9 k–10 k	~11.11%	(*43*)
	Graphene membrane	Electro-deformation	~13.4 M–16.4 M	~22.39%	(*44*)
	Dielectric electroactive polymer membranes (DEAP)	EAP strain tuning under kilovolt actuation	372.6–1620	334.78%	(*45*)
	Dielectric elastomer actuator (DEA) petal absorber	DEA petal bending driven under kilovolt actuation	697–1204	72.74%	(*46*)
	Magnetic membrane resonator	Magnetic tension tuning	86.63–88.73	2.4%	(*47*)
	Active poly (vinyl alcohol) /graphene (PVA/GR) membrane	Electric-field tuning of composite membrane	369–420	13.6%	(*48*)
	Metasurface	Manual tuning via screw-depth spiral channels	3.9–6.3 k	44.87%	(*49*)
	Tunable Helmholtz resonator	Continuous tuning via rigid neck-area control (iris valve)	~260–340	~24%	(50)
	Self-tuning side-branch Helmholtz resonator	Variable effective cavity volume and	~60–180	~200%	(51)

	tuning law for narrow-band tonal noise			
Electromechanical Helmholtz resonator	Electrical impedance loading of a piezo-backed rigid liner to vary effective acoustic impedance	band-dependent (tested 1–6.4 k)	~8–over 17%	(52)
**RAGSD (This work)**	**Pneumatic cavity tuning via integrated gas-driven module**	**922–1763**	**91.18%**	**This work**

Table S3. Adiabatic indices γ of air, He, CO_2, and N_2 at various temperatures (*81, 82*).

Gas	Temperature	γ
Air	0°C	1.403
	20°C	1.400
	100°C	1.401
	200°C	1.398
	400°C	1.393
	1000°C	1.365
Helium (He)	20°C	1.660
Carbon Dioxide (CO_2)	0°C	1.310
	20°C	1.300
	100°C	1.281
	400°C	1.235
Nitrogen (N_2)	-181°C	1.470
	15°C	1.404

Table S4. Comparison of other performances between the commercial electronic stethoscope and the RAGSD (*80*).

**Category**	**RAGSD (This work)**	**ETZ-1 (Commercial electronic stethoscope)**
Cost (dollar)	1.27	31.48
Weight (g)	6	110
Auscultation probe diameter (mm)	32	47
Probe construction	Soft and user-friendly	Hard

Table S5. Pseudocode representation of the AuscNet-H deep learning algorithm.

Algorithm 1: AuscNet-H Deep Learning Algorithm

```
Input: Input mel-spectrogram X ∈ R^{128×400×3}
Output: Predicted class logits or confusion matrix
Function InitializeNetwork():
    Initialize batch normalization layer BN_0 with 128 channels;
    Create 4 convolution blocks:
        ConvBlock(C_1): in=3, out=64;
        ConvBlock(C_2): in=64, out=128;
        ConvBlock(C_3): in=128, out=256;
        ConvBlock(C_4): in=256, out=512;
    Initialize fully-connected layer FC : 512 → 4;
Function ForwardPass(X):
    Reshape and permute: X ← Transpose(View(X));
    Apply BatchNorm: X ← BN_0(X);
    for i = 1 to 4 do
        X ← C_i (X) with pool size = (2,2), type = 'max';
        Apply Dropout with p = 0.2;
    Global Max Pooling: X ← MaxPool(X, kernel = X_spatial);
    Flatten: X ← View(X);
    Output logits: ŷ ← LogSoftmax(FC(X));
    return ŷ
Function TrainModel(TrainSet D_train):
    Load train dataset with batch size = 32, apply delta and normalization;
    Use MinimumOccupancySampler to balance classes in each mini-batch;
    Split training and validation set with validation split = 0.1;
    InitializeNetwork();
    Set optimizer: Adam (lr=0.0005, weight decay=0.0001, amsgrad=true);
    Set scheduler: StepLR (step size=20, gamma=0.9);
    Set loss: cross-entropy loss;
    Monitor: validation accuracy
    for epoch = 1 to 40 do
        foreach mini-batch (X, y) in training set do
            ŷ ← ForwardPass(X);
            Compute loss L ← CE(ŷ , y)
            Backpropagate and update weights;
        Validate on validation set and compute accuracy;
        Update learning rate using scheduler;
        if tensorboard enabled then
            Log training/validation metrics;
        if epoch % save period == 0 then
            Save model checkpoint;
    return Trained AuscNet-H model
Function Inference(TestSet D_test):
    Load best-performing model from saved checkpoint;
    Load test dataset;
    Construct test DataLoader with batch size and preprocessing;
    foreach mini-batch (X_test, y_test) in D_test do
        ŷ_test ← ForwardPass(X_test);
        Accumulate predictions and ground truth;
    Compute confusion matrix from predicted and true labels;
    Plot confusion matrix as heatmap;
    return Confusion matrix figures
Main: TrainModel(D_train); Inference(D_test);
```

Table S6. Summary of key symbols and their definitions used in the DCT-EMA model.

Symbol	Unit	Definition
$\mathbf{Z_m}$	N×s/m	Impedance of the mechanical oscillator
$\mathbf{R_m}$	N×s/m	Mechanical damping resistance of diaphragm
$\mathbf{C_m}$	m/N	Mechanical compliance of diaphragm
$\mathbf{k_m}$	N/m	Elastic coefficient of diaphragm (inverse of C_m)
$\mathbf{M_m}$	kg	Mechanical mass of diaphragm
ρ_e	kg/m^3	Density of Ecoflex 00-30
$\mathbf{r_0}$	m	Radius of the diaphragm
$\mathbf{l_0}$	m	Thickness of the diaphragm
$\mathbf{Z_a}$	$N \times s/m^5$	Acoustic radiation impedance
$\mathbf{Z_{a1}}$	$N \times s/m^5$	Internal radiation impedance of the membrane
$\mathbf{Z_{a2}}$	$N \times s/m^5$	External radiation impedance of the membrane
$\mathbf{R_a}$	$N \times s/m^5$	Acoustic radiation resistance
$\mathbf{M_a}$	kg/m^4	Acoustic mass of air loading
$\mathbf{C_a}$	m^5/N	Acoustic compliance of cavity
γ	–	Adiabatic index of gas (Table S1)
$\mathbf{P_g}$	Pa	Gas pressure in the cavity
$\mathbf{V_0}$	m^3	Initial cavity volume
$\mathbf{\Delta V}$	m^3	Injected gas volume
$\mathbf{R_{s1}}$	m	Equivalent spherical radius when $R_s<0$
$\mathbf{R_{s2}}$	m	Equivalent spherical radius when $R_s>0$
$\mathbf{R_s}$	m	Equivalent spherical radius
$\mathbf{h_1}$	m	Cap height when $R_s<0$
$\mathbf{h_2}$	m	Cap height when $R_s>0$
$\mathbf{Z_{total}}$	$N \times s/m^5$	Total impedance of the coupled circuit
$\mathbf{L, M_{total}}$	kg/m^4	Total equivalent mass
$\mathbf{C, C_{total}}$	m^5/N	Total equivalent compliance
$\mathbf{S}$	m^2	Equivalent area of diaphragm
$\mathbf{f}$	Hz	Resonant frequency

Table S7. Resonance tuning data under varying membrane dimensions and inflation gases.

ID	Diameter (mm)	Thickness (mm)	Gas	a_0	a_1	a_2	a_3	a_4	a_5
**1**	32	0.5	Air	348.289	-5.668	2.398E14	-1.963E19	7.865E23	-1.816E28
**2**	32	1.0	Air	143.029	-2.972E8	1.921E14	-1.675E19	6.995E23	-1.671E28
**3**	32	0.2	Air	-184.498	3.545E7	1.436E14	-1.526E19	7.307E23	-1.960E28
**4**	20	0.5	Air	2089.705	-4.141E9	2.652E15	-5.803E20	6.693E25	-4.591E30
**5**	26	0.5	Air	-664.703	3.592E8	2.316E14	-4.407E19	3.487E24	-1.514E29
**6**	32	0.5	He	1075.963	-7.835E8	2.878E14	-2.471E19	1.066E24	-2.697E28
**7**	32	0.5	N_2	276.390	-3.717E8	1.750E14	-1.256E19	3.991E23	-5.967E27
**8**	32	0.5	CO_2	325.077	-4.438E8	1.972E14	-1.495E19	5.268E23	-9.847E27

ID	Diameter (mm)	Thickness (mm)	Gas	a_6	a_7	a_8	a_9	R^2
**1**	32	0.5	Air	2.541E32	-2.130E36	9.859E39	-1.939E43	0.999
**2**	32	1.0	Air	2.406E32	-2.067E36	9.773E39	-1.957E43	1.000
**3**	32	0.2	Air	3.130E32	-2.956E36	1.523E40	-3.302E43	1.000
**4**	20	0.5	Air	1.942E35	-4.978E39	7.099E43	-4.326E47	1.000
**5**	26	0.5	Air	3.880E33	-5.857E37	4.820E41	-1.669E45	0.998
**6**	32	0.5	He	4.183E32	-3.927E36	2.050E40	-4.576E43	0.999
**7**	32	0.5	N_2	2.225E31	5.040E35	-6.616E39	2.424E43	0.999
**8**	32	0.5	CO_2	9.269E31	-2.549E35	-2.133E39	1.304E43	0.985

Supplementary Movies

Movie S1. Application of the RAGSD for personalized sound emission. This video demonstrates the real-time acoustic output of the RAGSD during continuous inflation from 0 to 20 mL. The device is driven by an AC-modulated signal derived from the chorus segment of *Opera No. 2*. As the cavity volume increases, the resonant frequency dynamically shifts. The perceived loudness of the singing voice first increases, then decreases, and is finally replaced by broadband noise. This corresponds to the resonance aligning with the main vocal frequency (approximately 1-1.1 kHz) at around 12 mL inflation (Fig. S7A). The result highlights the device's capacity for continuous and precise resonance tuning, enabling frequency-selective amplification in real time. Such personalized sound modulation offers potential for voice assistance applications, particularly for individuals with speech impairments.

Movie S2. Micro-pump operation for wearable pneumatic control. The video shows a benchtop setup with a control PCB driving a miniature air pump connected via flexible tubing. Playback is accelerated 20×. The sequence first presents the inflation state, where the pump delivers airflow through the tubing toward the downstream line, and then the deflation state, where the line is released to atmosphere.

Movie S3. Application of the RAGSD for intelligent wearable stethoscope. This video demonstrates the wearable use of RAGSD in a hospital environment. The packaged device is attached to the chest of a patient diagnosed with aortic regurgitation at the Seventh Affiliated Hospital of Sun Yat-sen University. After stable fixation, the device records cardiac sounds continuously for 1 min while the patient remains at rest. The recorded signals provide direct clinical data for evaluating pathological murmurs. The demonstration highlights the feasibility of applying RAGSD as an intelligent wearable stethoscope for real-world auscultation and data acquisition, complementing the controlled experimental validations presented in the main text.

Movie S4. Wearable RAGSD heart-sound sensing in a quiet environment. A volunteer wears the RAGSD chest-mounted device while seated at rest in an anechoic chamber. The movie shows real-time acquisition and display of heart sounds with the same processing pipeline as in the main text (continuous waveform and spectral view; identical gain and filter settings). No external noise is introduced. Time is real time.

Movie S5. Wearable RAGSD heart-sound sensing under added ambient noise. The same setup as Movie S3 is used, except that broadband environmental noise is played back in the chamber to create a noisy condition. The movie shows real-time acquisition and display with the same processing as in the main text (continuous waveform and spectral view; identical gain and filter settings). Time is real time.

Movie S6. On-body wearable auscultation across postures. This compilation presents three 5 s segments recorded with the full wearable RAGSD stethoscope system under non-anechoic indoor conditions. The subject remains instrumented with identical mounting and acquisition settings throughout. The sequence proceeds as seated, standing, and slow walking. Each segment shows real-time heart-sound acquisition with the same processing pipeline as in the main text (continuous waveform and spectrogram; identical gain and filter settings). Waveforms remain stable and heart-sound features are visible across postures, indicating robust coupling and practical usability during posture and ambient changes.

REFERENCES

1. G. Kirchhoff, Ueber den Einfluss der Wärmeleitung in einem Gase auf die Schallbewegung. *Ann. Phys.* **210**, 177–193 (1868).

2. N. Rott, Thermoacoustics. *Adv. Appl. Mech.* **20**, 135–175 (1980).

3. P. Novotný, T. Vít, M. Vestfálová, J. Lopes, Standing-wave thermoacoustic engines. *EPJ Web Conf.* **25**, 01061 (2012).

4. Y. Qiao, G. Gou, F. Wu, J. Jian, X. Li, T. Hirtz, Y. Zhao, Y. Zhi, F. Wang, H. Tian, Y. Yang, T.-L. Ren, Graphene-based thermoacoustic sound source. *ACS Nano* **14**, 3779–3804 (2020).

5. W. H. Preece, On some thermal effects of electric currents. *Proc. R. Soc. Lond.* **30**, 408–411 (1880).

6. T. Y. Kim, C.-H. Park, N. Marzari, The electronic thermal conductivity of graphene. *Nano Lett.* **16**, 2439–2443 (2016).

7. S. Zhang, H. Wang, J. Liu, C. Bao, Measuring the specific surface area of monolayer graphene oxide in water. *Mater. Lett.* **261**, 127098 (2020).

8. D. G. Papageorgiou, I. A. Kinloch, R. J. Young, Mechanical properties of graphene and graphene-based nanocomposites. *Prog. Mater. Sci.* **90**, 75–127 (2017).

9. I. A. Ovid'Ko, Mechanical properties of graphene. *Rev. Adv. Mater. Sci.* **34**, 1–11 (2013).

10. Y. Liu, B. Xie, Z. Zhang, Q. Zheng, Z. Xu, Mechanical properties of graphene papers. *J. Mech. Phys. Solids* **60**, 591–605 (2012).

11. F. De Nicola, S. Sarti, B. Lu, L. Qu, Z. Zhang, A. Marcelli, S. Lupi, Graphene aerogels for ultrabroadband thermoacoustics. *Phys. Rev. Appl.* **14**, 024022 (2020).

12. L. H. Tong, S. K. Lai, C. W. Lim, Broadband signal response of thermo-acoustic devices and its applications. *J. Acoust. Soc. Am.* **141**, 2430–2439 (2017).

13. Z. Zhou, Y. Feng, M. Xu, J. Wang, X. Xu, C. Lim, Theory and experiment for 3D porous graphene foam thermoacoustic transducer. *J. Phys. D Appl. Phys.* **55**, 035303 (2021).

14. J. W. Suk, K. Kirk, Y. Hao, N. A. Hall, R. S. Ruoff, Thermoacoustic sound generation from monolayer graphene for transparent and flexible sound sources. *Adv. Mater.* **24**, 6342–6347 (2012).

15. C. S. Kim, K. E. Lee, J.-M. Lee, S. O. Kim, B. J. Cho, J.-W. Choi, Application of n-doped three-dimensional reduced graphene oxide aerogel to thin film loudspeaker. *ACS Appl. Mater. Interfaces* **8**, 22295–22300 (2016).

16. H. Kim, J.-H. Ahn, Graphene for flexible and wearable device applications. *Carbon* **120**, 244–257 (2017).

17. T. Das, B. K. Sharma, A. K. Katiyar, J.-H. Ahn, Graphene-based flexible and wearable electronics. *J. Semicond.* **39**, 011007 (2018).

18. L.-Q. Tao, H. Tian, Y. Liu, Z.-Y. Ju, Y. Pang, Y.-Q. Chen, D.-Y. Wang, X.-G. Tian, J.-C. Yan, N.-Q. Deng, T.-L. Ren, An intelligent artificial throat with sound-sensing ability based on laser induced graphene. *Nat. Commun.* **8**, 14579 (2017).

19. H. Tian, Y. L. Cui, Y. Yang, D. Xie, T. L. Ren, "Wafer-scale flexible graphene loudspeakers" in *Proceedings of the 2014 IEEE 27th International Conference on Micro Electro Mechanical Systems (MEMS)* (IEEE, 2014), pp. 556–559.

20. H. Tian, C. Li, M. A. Mohammad, Y.-L. Cui, W.-T. Mi, Y. Yang, D. Xie, T.-L. Ren, Graphene earphones: Entertainment for both humans and animals. *ACS Nano* **8**, 5883–5890 (2014).

21. H. Tian, Y. Yang, D. Xie, T.-L. Ren, Y. Shu, C.-J. Zhou, L.-Q. Tao, L.-T. Liu, "Flexible and large-area sound-emitting device using reduced graphene oxide" in *Proceedings of the 2013 IEEE 26th International Conference on Micro Electro Mechanical Systems (MEMS)* (IEEE, 2013), pp. 709–712.

22. W. Hou, Y. Wei, Y. Wang, S. Duan, Z. Guo, H. Tian, Y. Yang, T.-L. Ren, A large-scale and low-cost thermoacoustic loudspeaker based on three-dimensional graphene foam. *ACS Appl. Mater. Interfaces* **16**, 23544–23552 (2024).

23. H. Tian, W. Gu, X.-S. Li, T.-L. Ren, Stretchable ink printed graphene device with weft-knitted fabric substrate based on thermal-acoustic effect. *ACS Appl. Mater. Interfaces* **15**, 20334–20345 (2023).

24. Y. Wei, Y. Qiao, G. Jiang, Y. Wang, F. Wang, M. Li, Y. Zhao, Y. Tian, G. Gou, S. Tan, H. Tian, Y. Yang, T. L. Ren, A wearable skinlike ultra-sensitive artificial graphene throat. *ACS Nano* **13**, 8639–8647 (2019).

25. H. Chen, F. Zhuo, J. Zhou, Y. Liu, J. Zhang, S. Dong, X. Liu, A. Elmarakbi, H. Duan, Y. Fu, Advances in graphene-based flexible and wearable strain sensors. *Chem. Eng. J.* **464**, 142576 (2023).

26. D. Shahdeo, A. Roberts, N. Abbineni, S. Gandhi, Graphene based sensors. *Compr. Anal. Chem.* **91**, 175–199 (2020).

27. Z. Yang, Y. Pang, X.-l. Han, Y. Yang, J. Ling, M. Jian, Y. Zhang, Y. Yang, T.-L. Ren, Graphene textile strain sensor with negative resistance variation for human motion detection. *ACS Nano* **12**, 9134–9141 (2018).

28. L. Chen, M. Weng, P. Zhou, F. Huang, C. Liu, S. Fan, W. Zhang, Graphene-based actuator with integrated-sensing function. *Adv. Funct. Mater.* **29**, 1806057 (2019).

29. Y. Qiao, X. Li, T. Hirtz, G. Deng, Y. Wei, M. Li, S. Ji, Q. Wu, J. Jian, F. Wu, Y. Shen, H. Tian, Y. Yang, T.-L. Ren, Graphene-based wearable sensors. *Nanoscale* **11**, 18923–18945 (2019).

30. H. Guo, J. Liu, H. Liu, M. Yang, J. Zhao, T. Lu, Iontronic dynamic sensor with broad bandwidth and flat frequency response using controlled preloading strategy. *ACS Nano* **18**, 5599–5608 (2024).

31. S. Gong, L. W. Yap, Y. Zhu, B. Zhu, Y. Wang, Y. Ling, Y. Zhao, T. An, Y. Lu, W. Cheng, A soft resistive acoustic sensor based on suspended standing nanowire membranes with point crack design. *Adv. Funct. Mater.* **30**, 1910717 (2020).

32. Y. Cotur, M. Kasimatis, M. Kaisti, S. Olenik, C. Georgiou, F. Güder, Stretchable composite acoustic transducer for wearable monitoring of vital signs. *Adv. Funct. Mater.* **30**, 1910288 (2020).

33. J. H. Han, J.-H. Kwak, D. J. Joe, S. K. Hong, H. S. Wang, J. H. Park, S. Hur, K. J. Lee, Basilar membrane-inspired self-powered acoustic sensor enabled by highly sensitive multi tunable frequency band. *Nano Energy* **53**, 198–205 (2018).

34. J. Park, D.-h. Kang, H. Chae, S. K. Ghosh, C. Jeong, Y. Park, S. Cho, Y. Lee, J. Kim, Y. Ko, J. J. Kim, H. Ko, Frequency-selective acoustic and haptic smart skin for dual-mode dynamic/static human-machine interface. *Sci. Adv.* **8**, eabj9220 (2022).

35. X. Zhao, Y. Zhou, A. Li, J. Xu, S. Karjagi, E. Hahm, L. Rulloda, J. Li, J. Hollister, P. Kavehpour, J. Chen, A self-filtering liquid acoustic sensor for voice recognition. *Nat. Electron.* **7**, 924–932 (2024).

36. Q. Zhang, Y. Wang, D. Li, J. Xie, R. Tao, J. Luo, X. Dai, H. Torun, Q. Wu, W. P. Ng, R. Binns, Y. Fu, Flexible multifunctional platform based on piezoelectric acoustics for human–machine interaction and environmental perception. *Microsyst. Nanoeng.* **8**, 99 (2022).

37. Y. Liu, H. Li, X. Liang, H. Deng, X. Zhang, H. Heidari, R. Ghannam, X. Zhang, Speech recognition using intelligent piezoresistive sensor based on polystyrene sphere microstructures. *Adv. Intell. Syst.* **5**, 2200427 (2023).

38. W. Qiao, L. Zhou, J. Zhang, D. Liu, Y. Gao, X. Liu, Z. Zhao, Z. Guo, X. Li, B. Zhang, Z. L. Wang, J. Wang, A highly-sensitive omnidirectional acoustic sensor for enhanced human–machine interaction. *Adv. Mater.* **36**, e2413086 (2024).

39. A. E. Aliev, D. H. Mueller, K. N. Tacker, N. K. Mayo, J. B. Blottman, S. Priya, R. H. Baughman, Improved thermoacoustic sound projectors by vibration mode modification. *J. Sound Vib.* **524**, 116753 (2022).

40. P. Kumar, R. Sriramdas, A. E. Aliev, J. B. Blottman, N. K. Mayo, R. H. Baughman, S. Priya, Understanding the low frequency response of carbon nanotube thermoacoustic projectors. *J. Sound Vib.* **498**, 115940 (2021).

41. E. J. Shin, S. Y. Park, C. S. Kim, B. J. Cho, J.-W. Choi, Construction of a multiway carbon nanotube loudspeaker with finely tunable resonance frequencies. *Adv. Mater. Technol.* **3**, 1700197 (2018).

42. Y.-H. Wei, Z.-F. Guo, Y.-F. Wang, T. Lin, W.-W. Hou, S.-W. Duan, L.-Q. Tao, H. Tian, Y. Yang, T.-L. Ren, Frequency-tunable sound amplification in a conch-like cavity with graphene thermoacoustic resonance. *Sci. Adv.* **11**, eadv2801 (2025).

43. J. I. Seeger, B. E. Boser, "Parallel-plate driven oscillations and resonant pull-in" in *Proceedings of the 2002 Solid-State, Actuators, and Microsystems Workshop* (University of California, 2002), pp. 313–316.

44. B. Sajadi, F. Alijani, D. Davidovikj, J. Goosen, P. G. Steeneken, F. van Keulen, Experimental characterization of graphene by electrostatic resonance frequency tuning. *J. Appl. Phys.* **122**, 234302 (2017).

45. P. Dubois, S. Rosset, M. Niklaus, M. Dadras, H. Shea, Voltage control of the resonance frequency of dielectric electroactive polymer (DEAP) membranes. *J. Microelectromechanical Syst.* **17**, 1072–1081 (2008).

46. M. Shrestha, G. K. Lau, Y. W. Chin, E. H. T. Teo, B. C. Khoo, Z. B. Lu, A tunable acoustic absorber using reconfigurable dielectric elastomer actuated petals. *Commun. Eng.* **3**, 11 (2024).

47. A. Gardiner, R. Domingo-Roca, J. F. C. Windmill, A. Feeney, An adjustable acoustic metamaterial cell using a magnetic membrane for tunable resonance. *Sci. Rep.* **14**, 15044 (2024).

48. Y. Li, S. Wang, Q. Peng, Z. Zhou, Z. Yang, X. He, Y. Li, Active control of graphene-based membrane-type acoustic metamaterials using a low voltage. *Nanoscale* **11**, 16384–16392 (2019).

49. S.-D. Zhao, A. L. Chen, Y.-S. Wang, C. Zhang, Continuously tunable acoustic metasurface for transmitted wavefront modulation. *Phys. Rev. Appl.* **10**, 054066 (2018).

50. Z. Zhang, D. Zhao, N. Han, S. Wang, J. Li, Control of combustion instability with a tunable Helmholtz resonator. *Aerosp Sci Technol* **41**, 55–62 (2015).

51. J. M. De Bedout, M. A. Franchek, R. J. Bernhard, L. Mongeau, Adaptive-passive noise control with self-tuning Helmholtz resonators. *J. Sound Vib.* **202**, 109–123 (1997).

52. F. Liu, S. Horowitz, G. Wang, T. Nishida, L. Cattafesta, M. Sheplak, "Characterization of a tunable electromechanical Helmholtz resonator" in *Proceedings of the 9th AIAA/CEAS Aeroacoustics Conference and Exhibit* (AIAA/CEAS, 2007), pp. 3145.

53. A. Purgue, paper presented at the Acoustical Society of America in 134th Meeting Lay Language Papers, San Diego, CA, 4 December 1997.

54. M. Gridi-Papp, The structure of vocal sounds produced with the mouth closed or with the mouth open in treefrogs. *J. Acoust. Soc. Am.* **123**, 2895–2902 (2008).

55. J. C. Case, E. L. White, R. K. Kramer, Soft material characterization for robotic applications. *Soft Robot.* **2**, 80–87 (2015).

56. Z. Liao, M. Hossain, X. Yao, Ecoflex polymer of different Shore hardnesses: Experimental investigations and constitutive modelling. *Mech. Mater.* **144**, 103366 (2020).

57. L. Jiang, Z. Fan, Design of advanced porous graphene materials: From graphene nanomesh to 3D architectures. *Nanoscale* **6**, 1922–1945 (2014).

58. R. Ye, D. K. James, J. M. Tour, Laser-induced graphene. *Acc. Chem. Res.* **51**, 1609–1620 (2018).

59. G. Bertuccio, On the physical origin of the electro-mechano-acoustical analogy. *J. Acoust. Soc. Am.* **151**, 2066–2076 (2022).

60. F. A. Firestone, A new analogy between mechanical and electrical systems. *J. Acoust. Soc. Am.* **4**, 249–267 (1933).

61. C. A. Nickle, Oscillographic solution of electromechanical systems. *Trans. Am. Inst. Electr. Eng.* **44**, 844–856 (1925).

62. L. L. Beranek, T. Mellow, *Acoustics: Sound fields and transducers* (Academic Press, 2012).

63. B. Koonce, "VGG network" in *Convolutional Neural Networks with Swift for Tensorflow: Image Recognition and Dataset Categorization*, B. Koonce, Ed. (Apress, 2021), pp. 35–50.

64. P. Kim, "Convolutional neural network" in *MATLAB Deep Learning: With Machine Learning, Neural Networks and Artificial Intelligence* (Springer, 2017), pp. 121–147.

65. M. Egmont-Petersen, D. de Ridder, H. Handels, Image processing with neural networks—A review. *Pattern Recognit.* **35**, 2279–2301 (2002).

66. G. Y. Yaseen, S. Son, S. Kwon, Classification of heart sound signal using multiple features. *Appl. Sci.* **8**, 2344 (2018).

67. Y. T. Suy, Information content of a sound spectrogram. *J. Audio Eng. Soc.* **15**, 407–413 (1967).

68. P. Spelda, V. Stritecky, Human induction in machine learning: A survey of the nexus. *ACM Comput Surv* **54**, 1–18 (2021).

69. A. Géron, Ed., *Hands-On Machine Learning with Scikit-Learn, Keras, and TensorFlow* (O'Reilly Media, 2022).

70. P. Xanthopoulos, P. M. Pardalos, T. B. Trafalis, "Linear discriminant analysis" in *Robust Data Mining*, P. Xanthopoulos, P. M. Pardalos, T. B. Trafalis, Eds. (Springer, ed. 1, 2013), pp. 27–33.

71. L. L. Beranek, Acoustics. *Phys. Today* **8**, 27–28 (1955).

72. J. R. Chang, C. N. Wang, Acoustical analysis of enclosure design parameters for microspeaker system. *J. Mech.* **35**, 1–12 (2019).

73. R. Liechti, Equivalent electrical circuits for electroacoustic MEMS design: A review. *J. Micromech. Microeng.* **34**, 083004 (2024).

74. T. Ten Wolde, Reciprocity measurements in acoustical and mechano-acoustical systems. Review of theory and applications. *Acta Acust. united Acust.* **96**, 1–13 (2010).

75. E. Huerta, J. E. Corona, A. I. Oliva, F. Avilés, J. González-Hernández, Universal testing machine for mechanical properties of thin materials. *Rev. Mex. Fis.* **56**, 317–322 (2010).

76. R. H. Small, Closed-box loudspeaker systems Part II: Synthesis. *J. Audio Eng. Soc.* **21**, 11–18 (1973).

77. R. H. Small, Closed-box loudspeaker systems Part I: Analysis. *J. Audio Eng. Soc.* **20**, 978–808 (1972).

78. R. Ger, On some properties of polynomial functions. *Ann. Pol. Math.* **25**, 195–203 (1971).

79. R. Janardhana, F. Akram, Z. Guler, A. Adaval, N. Jackson, A comprehensive experimental, simulation, and characterization mechanical analysis of Ecoflex and its formulation under uniaxial testing. *Materials* **18**, 3037 (2025).

80. Zibo Exagiga Electric Co. Ltd., Novel electronic stethoscope. China patent CN208973899U, 14 June 2019.

81. J. A. Dean, *Lange's Handbook of Chemistry* (McGraw-Hill, ed. 15, 1999).

82. F. M. White, J. Majdalani, *Viscous Fluid Flow* (McGraw-Hill, ed. 3, 2006).
